# Analysis of co-expression and gene regulatory networks associated with sterile lemma development in rice

**DOI:** 10.1186/s12870-022-04012-x

**Published:** 2023-01-06

**Authors:** Xi Luo, Yidong Wei, Yanmei Zheng, Linyan Wei, Fangxi Wu, Qiuhua Cai, Huaan Xie, Jianfu Zhang

**Affiliations:** 1 College of Agriculture, Fujian Agriculture and Forestry University, Fuzhou, 350002 China; 2 Rice Research Institute, Fujian Academy of Agricultural Sciences, Fuzhou, 350019 China; 3 Key Laboratory of Germplasm Innovation and Molecular Breeding of Hybrid Rice for South China, Ministry of Agriculture and Affairs P.R. China/Incubator of National Key Laboratory of Germplasm Innovation and Molecular Breeding between Fujian and Ministry of Sciences and Technology/Fuzhou Branch, National Rice Improvement Center of China/Fujian Engineering Laboratory of Crop Molecular Breeding/Fujian Key Laboratory of Rice Molecular Breeding, Fuzhou, 350003 China

**Keywords:** Rice, Sterile lemma, Regulatory network, Co-expression network, Machine learning, *G1*

## Abstract

**Background:**

The sterile lemma is a unique organ of the rice (*Oryza sativa* L.) spikelet. However, the characteristics and origin of the rice sterile lemma have not been determined unequivocally, so it is important to elucidate the molecular mechanism of the development of the sterile lemma.

**Results:**

In the paper, we outline the regulatory mechanism of sterile lemma development by *LONG STERILE LEMMA1* (*G1*), which has been identified as the gene controlling sterile lemma development. Based on the comprehensive analyses of transcriptome dynamics during sterile lemma development with *G1* alleles between wild-type (WT) and mutant (MT) in rice, we obtained co-expression data and regulatory networks related to sterile lemma development. Co-transfection assays of rice protoplasts confirmed that *G1* affects the expression of various phytohormone-related genes by regulating a number of critical transcription factors, such as *OsLBD37* and *OSH1*. The hormone levels in sterile lemmas from WT and MT of rice supports the hypotheses that lower auxin, lower gibberellin, and higher cytokinin concentrations are required to maintain a normal phenotype of sterile lemmas.

**Conclusion:**

The regulatory networks have considerable reference value, and some of the regulatory relationships exhibiting strong correlations are worthy of further study. Taken together, these work provided a detailed guide for further studies into the molecular mechanism of sterile lemma development.

**Supplementary Information:**

The online version contains supplementary material available at 10.1186/s12870-022-04012-x.

## Background

Rice (*Oryza sativa* L.) is probably the most important grain crop in the world. It is also the model plant for monocotyledons. The morphological structure of the inflorescence and spikelet has important effects on yield and quality of rice. However, compared with dicotyledons such as Arabidopsis (*Arabidopsis thaliana*), the progress made in our understanding of flower development has been much slower in rice. This is due to the complex structure of the flower organs and the absence of related mutants in monocotyledons. The spikelet is an inflorescence structure unique to the Poaceae, while, in addition the spikelet of rice has its own architecture. In rice each spikelet has only one floret and a pair of unique sterile lemmas, compared with other grasses.

Regarding the evolution and origin of the sterile lemmas, two hypotheses have been proposed to date. One suggests that the spikelet of a putative ancestor of *Oryza* contained three florets: a terminal floret and two lower lateral florets. The lateral florets degenerated during evolution, leaving only the lemmas, which subsequently degenerated into sterile lemmas [1, 2]. The other hypothesis is that the spikelet of *Oryza* always had only one floret, and that the sterile lemmas and rudimentary glumes are severely reduced bract structures [3]. Recently, a growing body of evidence has come to light which supports the former hypothesis [4, 5]. Ren believe that the integration of sterile lemma mutants of *G1*, *MFS1*, *FON4*, *EG1* and other genes with the three-flowered mutant *lf1* could produce a new rice ideotype with intact and fertile three-flowered spikelets [6, 7]. These findings have identified a new strategy by which to for potentially increase yield of rice.

There have been many reports on the morphological changes to the sterile lemmas, which are caused by mutations of different genes [8]. However, gene expression profiling and identification of the regulatory networks that control development of the sterile lemmas are yet to be achieved. In the current study, we conducted a detailed RNA-sequencing (RNA-Seq) analysis of the different stages of sterile lemma development in a mutant with the phenotype of long, sterile lemmas and its wild-type parent. We identified several gene sets related to sterile lemma development by weighted gene co-expression network analysis (WGCNA) and identified transcription factors in these gene sets. Furthermore, using the genes encoding these transcription factors as input genes, a regulatory network was constructed through a machine-learning approach. Among these regulatory relationships, we focused on the genes associated with biosynthesis or signal transduction of various phytohormones. Determination of the concentrations of phytohormones confirmed that differential expression of some key genes caused significant changes in the concentrations of the corresponding hormones. On the other hand, *LONG STERILE LEMMA1* (*G1*)*/ELONGATED EMPTY GLUME* (*ELE*) has been reported to be the identity gene of sterile lemma [9–11]. Our results showed that *G1/ELE* is involve in the regulatory pathways of several strong transcription factor candidates on the development of rice sterile lemma.

## Results

### Phenotypic comparison of the long, sterile lemma mutant and its wild type

**Fig. 1:**
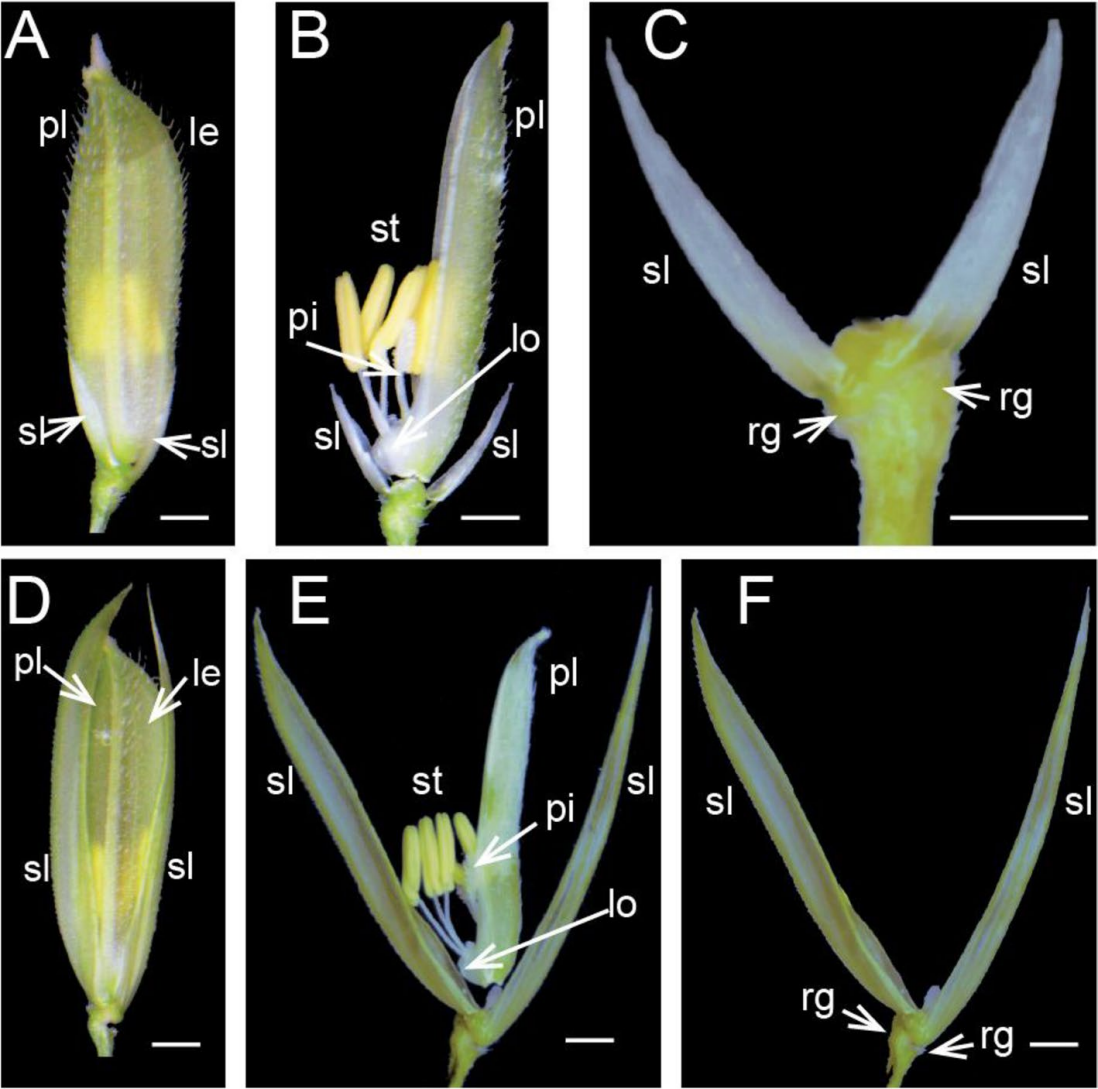
Phenotype of sterile lemmas of wild type and mutant rice at florescence. (**a**)-(**c**) Spikelet structure of wild type. (**d**)-(**f**) Spikelet structure of *g1–7* mutant. Sl: sterile lemma; le: lemma; pa: palea; lo: lodicules; st: stamen; pi: pistil. Scale bar = 1 mm

**Fig. 2:**
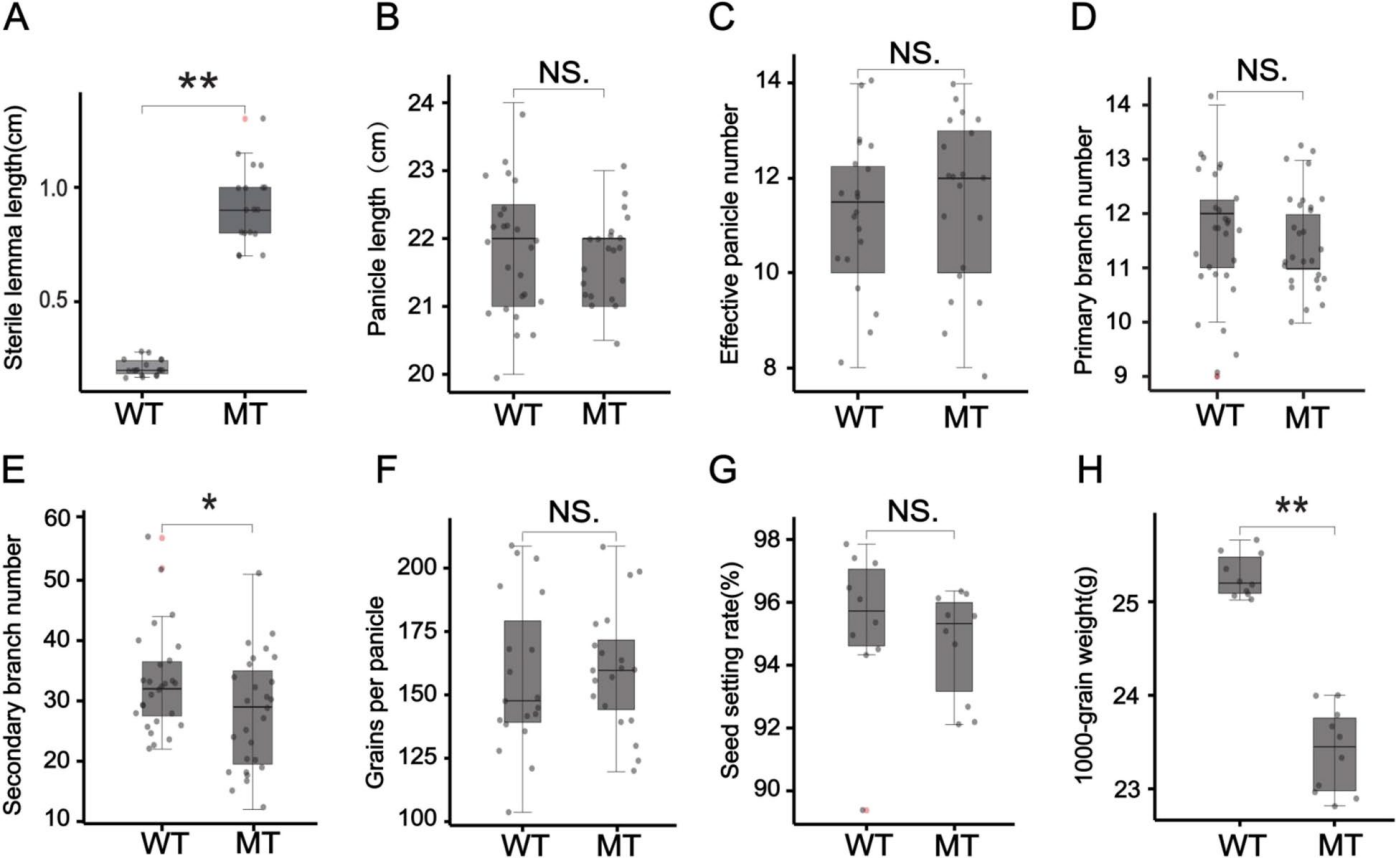
Box-and-whiskers comparison of yield-related traits between wild type (WT) and mutant (MT) with long, sterile lemmas. **a** Sterile lemma length. **b** Panicle length. **c** Effective panicle number. **d** Primary branch number. **e** Second branch number. **f** Grains per panicle. **g** Seed setting rate. **h** 1000-grain weight. A horizontal line in the middle of each box represents the median, and the upper and lower ends of each box represents the upper and lower quartiles, respectively. Each black dot represents a data point from the sample, whereas red dots represent the coordinates of any outliers. The whiskers indicate the minimum and maximum values, respectively. Two-tailed Student’s t-test was performed to determine the significance of any difference in each trait between wild type and mutant (NS, not significant;*, *P* < 0.05; **, *P* < 0.01)

We compared the morphology of spikelet organs at florescence between the mutant and wild type (Fig. 1). The morphology of spikelet organs such as the paleas, lemmas, and inner flower organs of mutant were not significantly different from the wild type except that the sterile lemma size of the mutant was significantly greater than that of the wild type (Fig. 2a). Considering that *TAWAWA1* [12], another gene in the ALOG family to which *G1* belongs, is associated with rice yield, we analyzed yield-related traits of the *G1* mutant and wild type at maturity (Fig. 2bh). Compared with the wild type, there was no significant difference in panicle length, effective panicle number, primary branch number, grains per panicle and seed setting rate of the mutant. However, the secondary branch number and 1000-grain weight of the mutant were significantly lower than those of the wild type. These results suggested that *G1* functions to suppress the overgrowth of the sterile lemmas, whereas the effect of controlling secondary branch number was similar to *TAWAWA1.*

### Analysis of differentially expressed genes by RNA-Seq

To reveal the transcriptional network underlying the development of sterile lemmas, we performed RNA-Seq analysis of young panicles at four stages and of sterile lemmas at the booting stage (Fig. S1). A previous study [13] had shown that sterile lemmas began to differentiate when panicles reached a length of 0.9–1.5 mm. In the current study, because of the technical difficulties associated with separating the tiny, sterile lemmas from their inflorescence, we sampled young panicles with a length of 1.0–15 mm for RNA-Seq (Table 1).

**Table 1:** Description of samples collected for RNA-Seq at different panicle development stages

Symbol	Sampling points	Length of young panicle	Apical spikelet development stage
S1	Young panicle	1mm~2mm	Formation of palea, elongation of sterile lemma and lemma
S2	Young panicle	2mm~5mm	Formation of stamen and pistil primordia
S3	Young panicle	5mm~10mm	Stamen and pistil begin to differentiate, palea and lemma close up gradually
S4	Young panicle	10mm~15mm	Formation of pollen and ovule
SL	Sterile lemma	—	Booting stage

**Fig. 3:**
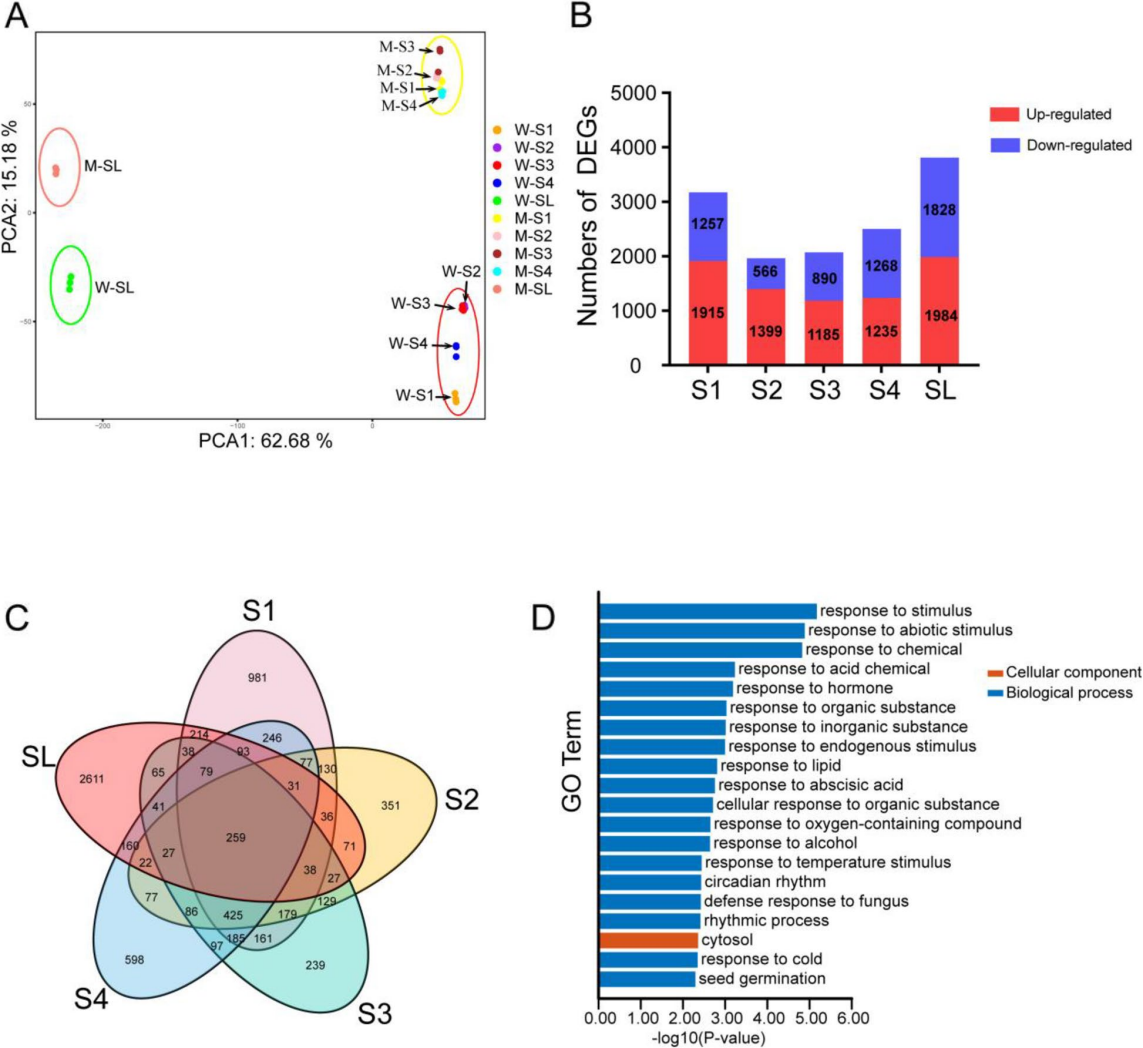
Analysis of differentially expressed genes (DEGs). (**a**) Principal component analysis of the RNA-Seq data. The letters ‘W’ and ‘M’ represent wild type and mutant type, respectively. (**b**) Numbers of differentially expressed genes (DEGs). The numbers in the bar graph are the number of up-regulated or down-regulated genes. (**c**) Venn diagram of DEGs among the five samples. (**d**) Top 20 significantly enriched Gene Ontology (GO) terms (*P*-value < 0.01) of the 259 DEGs overlapping in the five samples

In total, 30 cDNA libraries were constructed and sequenced. The RNA-Seq data were uploaded to the Sequence Read Archive (SRA) of the National Center for Biotechnology Information (accession number PRJNA819495;). The results of RNA-Seq showed that 35,666 genes were expressed in different samples. Principal component analysis (PCA) revealed that the ten samples could be clearly assigned to four groups. The transcriptome profiling of the mutant and the wild type were obviously clustered into two groups, whether in young panicles or in sterile lemmas (Fig. 3a). The PCA results showed the RNA-Seq data had obvious clustering characteristics and good intergroup repeatability, so could be used for further analyses.

We regarded the genes where |log2 (fold change)| > 1 between the two genotypes to be differentially expressed genes (DEGs). Overall, there were more up-regulated genes than down-regulated genes in each sample, During the development of sterile lemmas, the number of DEGs decreased at stage S2 (relative to stage S1) first and then it increased at stages S3, S4, SL (Fig. 3b). Compared with the other samples, the number of DEGs in SL was the highest.

To narrow down the range of candidate DEGs associated with the development of sterile lemmas, we identified 259 DEGs that were detected in the five stages sampled (Fig. 3c). We drew the heat map with fold change of the 259 DEGs. After clustering, we found that 138 DEGs were upregulated in the MT relative to the WT, and 82 DEGs were downregulated; 34 DEGs were first upregulated and then downregulated, while five DEGs showed the opposite pattern (Fig. S2). The GO enrichment analysis was performed to annotate the 259 DEGs which intersection of the five samples (Fig. 3d). The results showed that nineteen Gene Ontology (GO) terms were related to biological processes whereas only one term was related to cell components in the top 20 GO terms with significant enrichment. We focused on the GO terms that were associated with response to hormone. There are 26 genes in this GO term (Table S1), the description of these genes including auxin-responsive promoter, a variety of enzymes, transcription factors and so on. This result implied that the hormone level in the mutant is likely to be altered relative to the wild type.

### Screening of modules related to sterile lemma development and co-expression network construction

**Fig. 4:**
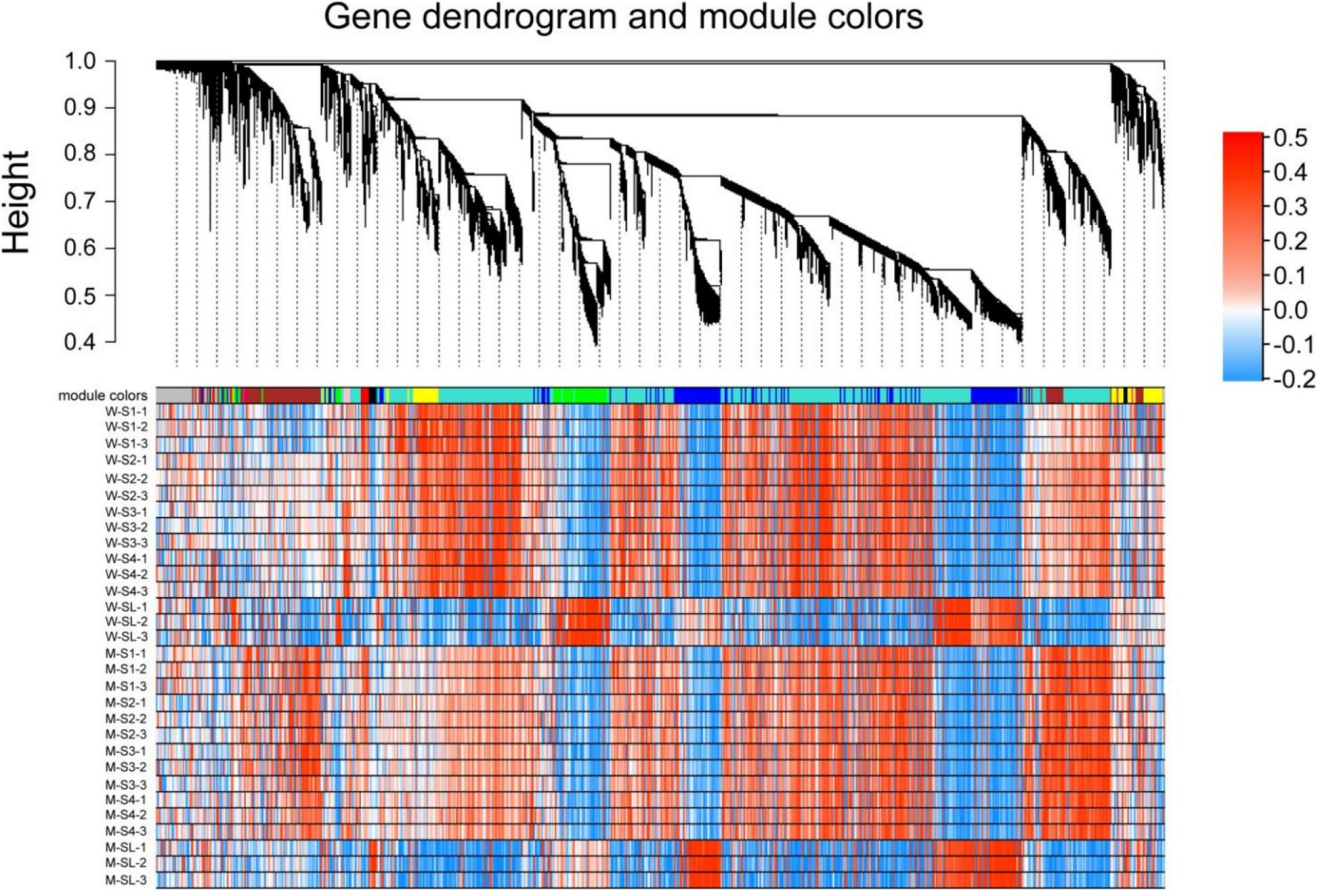
Gene dendrogram showing co-expression modules identified by WGCNA. Each leaf in the tree represents one gene, and the heat map show the expression level of the gene in different samples; The expressed values in the heat map were transformed by log10 (FPKM+ 1). The nine modules were labeled with different colors, with gray representing genes that are not clustered into any module. The letters ‘W’ and ‘M’ represent wild type and mutant type respectively. The numbers ‘1’, ‘2’, ‘3’ represent the three biological duplications of each sample

To identify regulatory networks which are associated with the development of sterile lemmas in rice, a gene co-expression network analysis of the 35,666 genes expressed across the five stages was performed using the WGCNA package [14]. Because the biological samples in this study have obvious clustering characteristics and the average connectivity of the clustered genes is high, therefore we chose a higher value of soft threshold for topology matrix (TOM) calculation (Fig. S3). Based on the TOM value matrix, we clustered the expressed genes by the dynamic mixed cutting method. We obtained nine gene sets (hereafter referred to as ‘modules’) where genes have co-expression patterns **(**Fig. 4**)**.

**Fig. 5:**
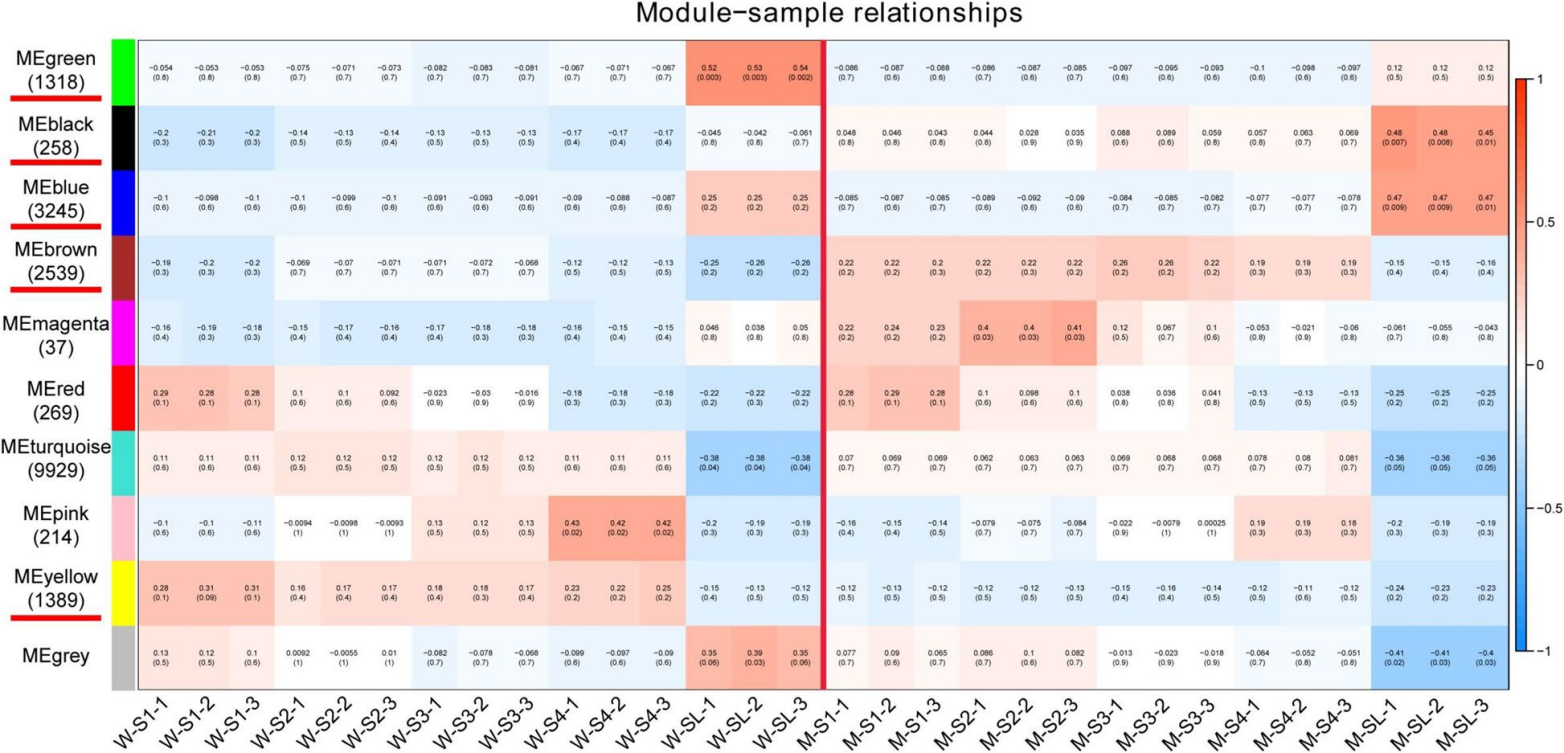
Correlation analysis between eigengenes and samples. The numbers in brackets indicate the number of genes in the module. WT: wild type; MT: mutant type. The number in the first line of the cell is the correlation coefficient, and the second line is the *P*-value

**Fig. 6:**
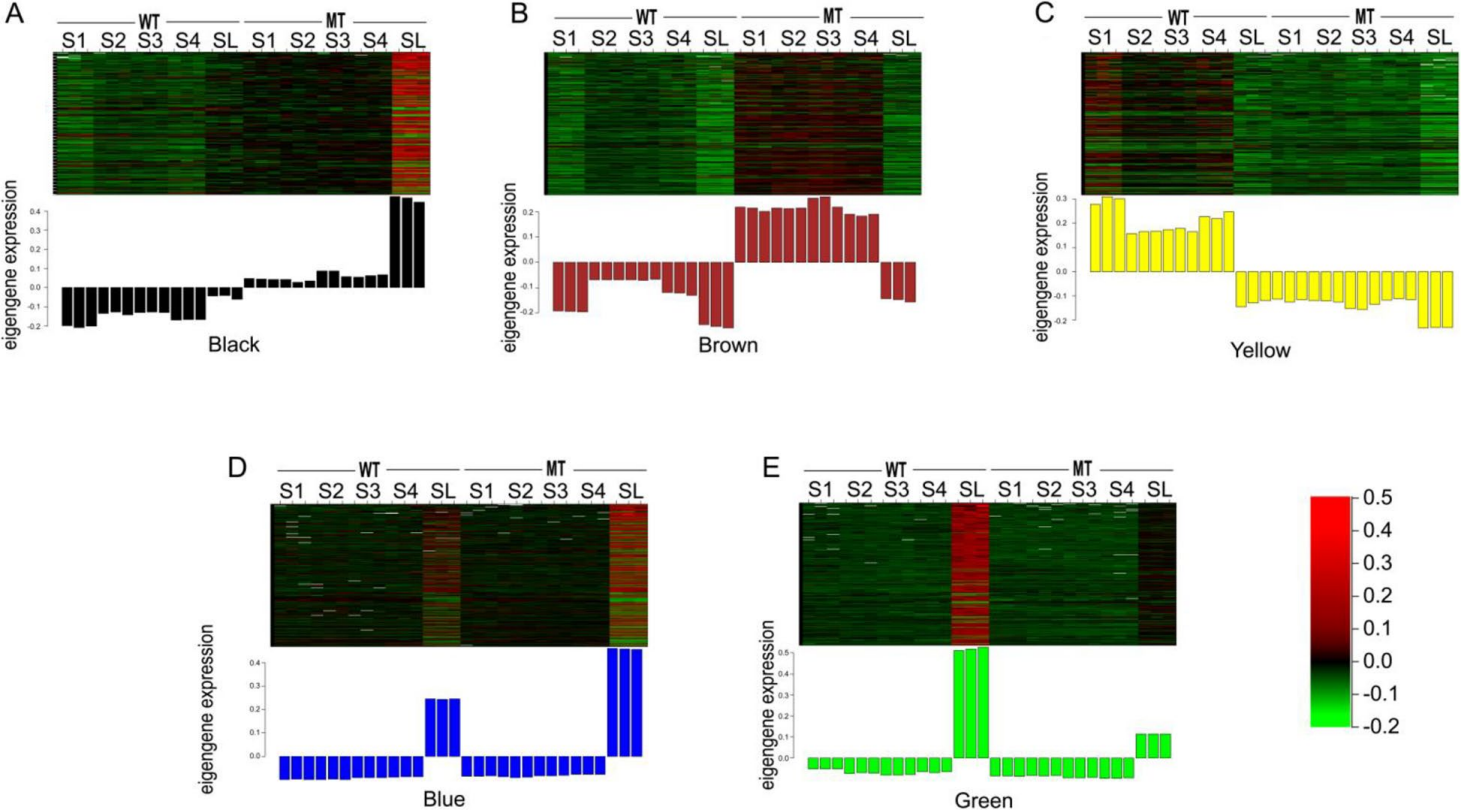
Expression heatmap of module genes in representing five stages of panicle development samples. Each row represents one gene and each column represents a biological replicate of the corresponding sample. The expressed values in the heat map are transformed by log_10_ (FPKM+ 1). WT: wild type; MT: mutant type; Bar chart: expression level of eigengenes. **a** The expression of genes assigned to the black module. **b** The expression of genes assigned to the brown module. **c** The expression of genes assigned to the yellow module. **d** The expression of genes assigned to the blue module. **e** The expression of genes assigned to the green module

When we conducted the correlation analysis between modules and samples. Seven out of the nine modules were specific to the long, sterile lemma phenotype in the different developmental stages (Fig. 5)**.** The black module contained 258 genes specific to the long, sterile lemma phenotype across all five developmental stages. Most of the genes in the black module were upregulated in each sample of the mutant relative to the wild type **(**Fig. 6a**)**. The brown and yellow modules identified 2539 and 1389 genes, respectively, which were specific to the long, sterile lemma phenotype across four developmental stages of the young panicle **(**Fig. 5**)**. Most of the genes in the brown module were upregulated at four stages of the mutant type (Fig. 6b), whereas those in the yellow module showed the opposite expression pattern **(**Fig. 6c**)**. Blue and green modules contained 3245 and 1318 genes respectively **(**Fig. 5**)**, specific to the phenotype of the long, sterile lemmas at the booting stage. Most of the genes in the blue module were upregulated in the long, sterile lemmas of the mutant (Fig. 6d), whereas in the green module showed the opposite expression pattern (Fig. 6e). Magenta and pink modules contained genes specific to the long, sterile lemma phenotype at stages S1 and S2 and at stages S3 and S4, respectively **(**Fig. 5**)**. In order to ensure that the key genes controlling the development of the sterile lemma can be screened and to simplify the analytical process, five modules (modules marked with red lines in Fig. 5) that showed differential expression across multiple developmental stages or only in the sterile lemmas at the booting stage were selected for further analysis.

Next, we visualized the gene co-expression network for each module based on the results of WGCNA. GO enrichment analysis was performed on genes in each module (Figs. S4–S8). The genes with top 10 degree value were selected as the HUB genes, and the description information, number of significant GO terms, research progress and other known information of the ten HUB genes were summarized (Tables S2–S6). We found several HUB genes that were involved in either plant growth or development, such as the aldehyde dehydrogenase family genes *OsALDH6B2*, *OsALDH2B5* [15–17] and indoleacetic amino synthase gene *OSGH3–1* [18, 19] in the black module, and the phytohormone balance gene *PLA3/GO* [20] in the brown module. The plant development regulator gene *HOX9* [21] and histidine phosphate transfer protein gene *OsAHP2/OHP2* [22, 23] in the yellow module and the transcription factor gene *OsNAC2* [24, 25] in the blue module can regulate auxin, cytokinin and gibberellin signaling pathway-related genes; The *OsSUT1* gene [26, 27] encoding a sucrose transporter and two small auxin-up RNAs (SAURs) genes that code for auxin-responsive were identified in the green module. Most of the HUB genes mentioned above were also differentially expressed genes (|log2 (fold change)| > 1), indicating that the mutation of *G1* affected the expression of these genes, thereby further regulating the development of sterile lemma.

### Identification of transcription factors and construction of regulatory networks in modules related to sterile lemma development

First, we identified all the transcription factors in the five modules and found that the green module had the highest proportion of transcription factors (Table S7). We also calculated the proportion of the sum of the transcription factors which belong to a development-related family in the five modules to all members of each family in rice. The results showed that the proportion of GARP, bZIP, SBP and MYB families were higher in the modules related to sterile lemma development (Table S7).

To identify the determine target genes which are regulated by transcription factors in the five modules, we used the identified transcription factors as input genes, and the same sample set in WGCNA was used as expression matrix. GENIE3 machine-learning algorithm based on R package (GENIE3 1.18.0) was used to determine the regulatory relationships between the various transcription factors and their downstream target genes. In the top 500 links, there are 141 transcription factors that regulate 420 target genes, with more transcription factors in the blue, brown and green modules (41, 41 and 31, respectively) than in the black and yellow modules (6 and 22 respectively).

To obtain a general understanding of the 420 target genes, GO enrichment analysis was performed (Fig. S9). In the top 20 GO terms with significant enrichment, there were 19 terms related to the regulation of various biological processes, and one term related to the cell component ‘boundary membrane of organelles’. In these GO terms, we focused on the terms ‘flower development’, ‘plant organ formation’, ‘reproductive shoot system development’ and ‘regulation of developmental process’. We speculate that the genes included in these four GO terms are potentially involved in sterile lemma development. Therefore, we summarized the related descriptions of these genes (Table S8). These include several crucial genes that are known to regulate flower development in rice, such as the *OsGA20ox1* gene encoding a key oxidase enzyme in the biosynthesis of gibberellins, the spikelet meristem identity gene *FZP* (*FRIZZY PANICLE*) and the *YUCCA6* auxin (IAA) biosynthetic pathway gene.

**Fig. 7:**
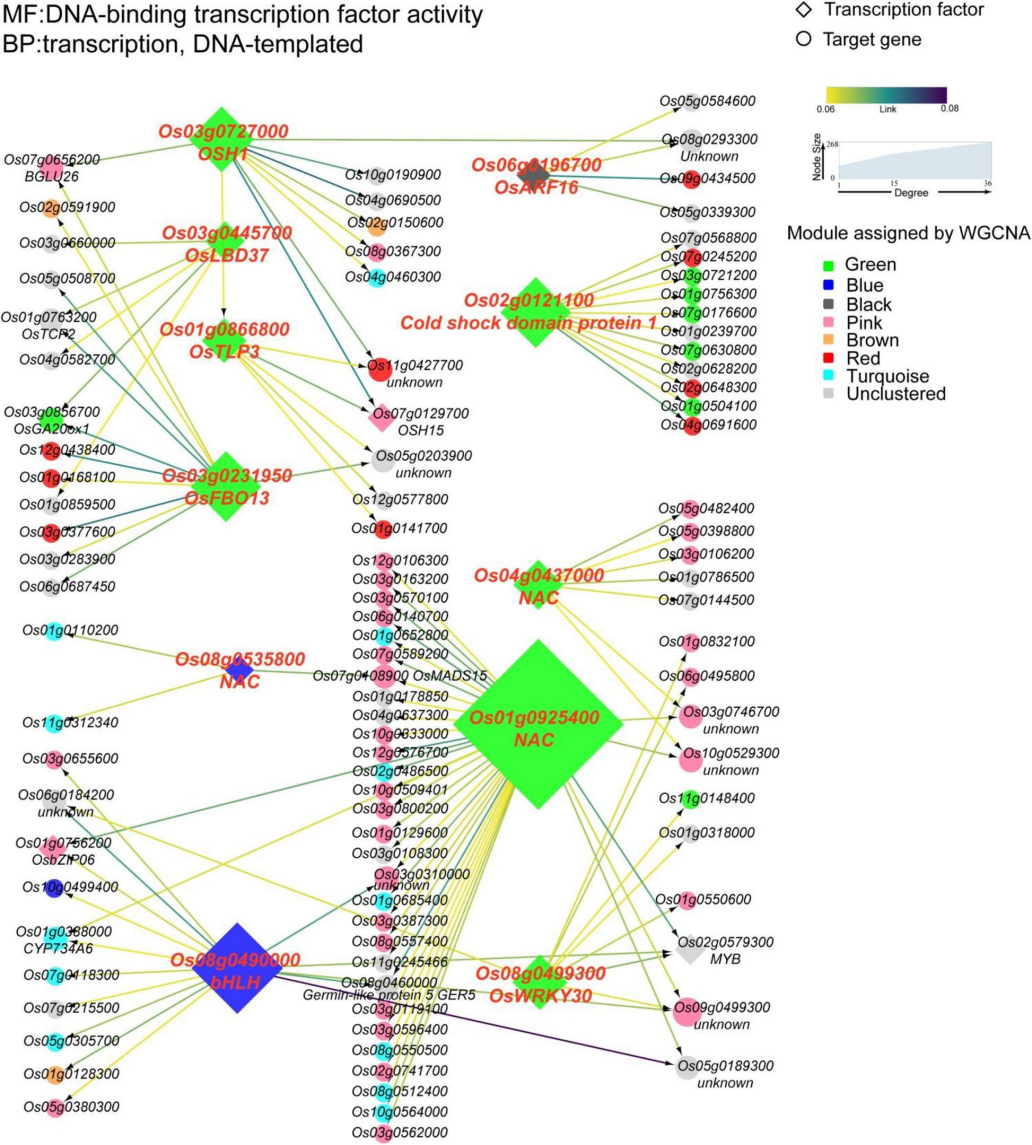
The first type of regulatory network. The annotations in the upper left corner are the GO terms that minimize the *P*-value in the enrichment analysis of all genes in the network; CC: cell components; MF: Molecular function; BP: Biological process. Transcription factors are annotated with symbols or family names; descriptions of target genes regulated by more than two transcription factors are annotated

Next, we visualized these regulatory relationships (Fig. S10), and we focused on three types of larger regulatory networks. The first type of regulatory network is centered on transcription factors in the green module. The target genes mainly belong to the pink, turquoise modules and to the genes that are not clustered to any module (Fig. 7). There are three NAC family transcription factors in the regulatory network, one (*Os01g0925400*) of which regulates 36 downstream target genes and is the transcription factor with the largest number of target genes in the whole regulatory network. In second type of regulatory network, *KNOX* (*Knotted1-like homeobox*) gene *OSH15* is predicted to be the target gene of *Oryza sativa homeobox1* (*OSH1*). A previous study had demonstrated that *OSH1* binds to five KNOX loci, including *OSH1* and *OSH15* directly, through conserved *cis*-elements, and that the positive autoregulation of *OSH1* is indispensable for its own expression and maintenance of the shoot apical meristem [28]. The OsLBD37 transcription factor is a member of the LATERAL ORGAN BOUNDARIES DOMAIN (LBD) family, which is a plant-specific protein containing representative LATERAL ORGAN BOUNDARIES (LOB) domains. Studies have shown that *OsLBD37*-overexpressed plants delayed heading stage by about 30 days and doubled the number of grains per panicle [29]. In this regulatory network, the GA20 oxidase gene *OsGA20ox1* is the target gene regulated by *OsLBD37*, and GA20 oxidase is a key enzyme that normally catalyzes the penultimate steps in GA biosynthesis [30]. *OSH1*, *OsLBD37* and *OsGA20ox1* play crucial roles in the formation and development of floral organs in rice, and these genes may also be involved in the development of sterile lemmas in rice.

The second type of regulatory network is centered on transcription factors in the blue module, and the target genes mainly belong to the red module, the turquoise module and the genes that are not clustered to any module (Fig. S11). *OsNAC2* in this network is also the HUB gene of the blue module. Previous studies have shown that *OsNAC2* functions as a negative regulator involved in the regulation of the gibberellin (GA) signaling pathway. On the other hand, *OsNAC2* functions as an upstream integrator inhibiting the activity of auxin and increasing the concentration of cytokinin [25]. In the present study, the expression level of *OsNAC2* was clearly decreased (fold change is − 1.07) in a sample of mutant at the S2 stage. In this network, *RAI1* and *OsMADS13* regulate the grain weight gene *GW6a* [31], and the link value is higher, indicating that these two transcription factors are closely related to *GW6a*. In this network, three MYB transcription factors co-regulate some genes with *OsNAC2* and GARP-G2-like transcription factors, indicating that these transcription factors play a synergistic role in the same regulatory network. *FZP* belongs to the EREBP family of the AP2 superfamily and is considered to be the identity gene of spikelet meristems [32, 33]. In the current study, only a low abundance of *FZP* transcripts was detected in stages S1 and S2. The prediction result showed that *FZP* is the target gene of *OsARF25* [34, 35] and *OsARF4* [36, 37] related to IAA signal transduction, indicating that *FZP* is involved in the regulation of IAA concentration at the early stage of young panicle development.

The third type of regulatory network is centered on transcription factors in the brown and yellow modules, and most of the target genes are also genes in the brown and yellow modules. In this regulatory network, we note that two AP2 transcription factors and two MYB transcription factors share a common target gene, namely *Os05g0417900* (Fig. S12). The function of the homologous gene of *Os05g0417900* in maize (*Zea mays*) or broomcorn millet (*Panicum miliaceum*) is as an auxin-inducible protein related to organ size [38, 39]. In the present study, the expression of this gene was upregulated at stages S1, S2, S3 and S4 with fold changes of 0.15, 1.55, 0.84 and 1.31 respectively, and downregulated (fold change is − 2.02) in the sterile lemma at booting stage of the mutant compared with the wild type. The results implied that the content of auxin in the mutant may be changed and affect the expression level of *Os05g0417900*.

### Identification of phytohormone-related genes in the green and blue modules

The levels of expression of the genes in the green and blue modules were significantly correlated with the sterile lemmas phenotype (Fig. 6d, e). Meanwhile, identification of transcription factors had revealed a higher proportion of transcription factors in these two modules than in the others (Table S7). GO enrichment analysis showed that most of the target genes of these transcription factors were related to plant development process and some genes were involved in the metabolic regulation of phytohormones. To determine the genes that were involved in phytohormone synthesis or signaling pathways, we identified the phytohormone genes in the two modules and located these genes in the metabolic pathways which involved phytohormones, including IAA, CKs or GAs. We also took account of the expression heatmaps of the corresponding genes in sterile lemmas of the mutant and the wild type.

**Fig. 8:**
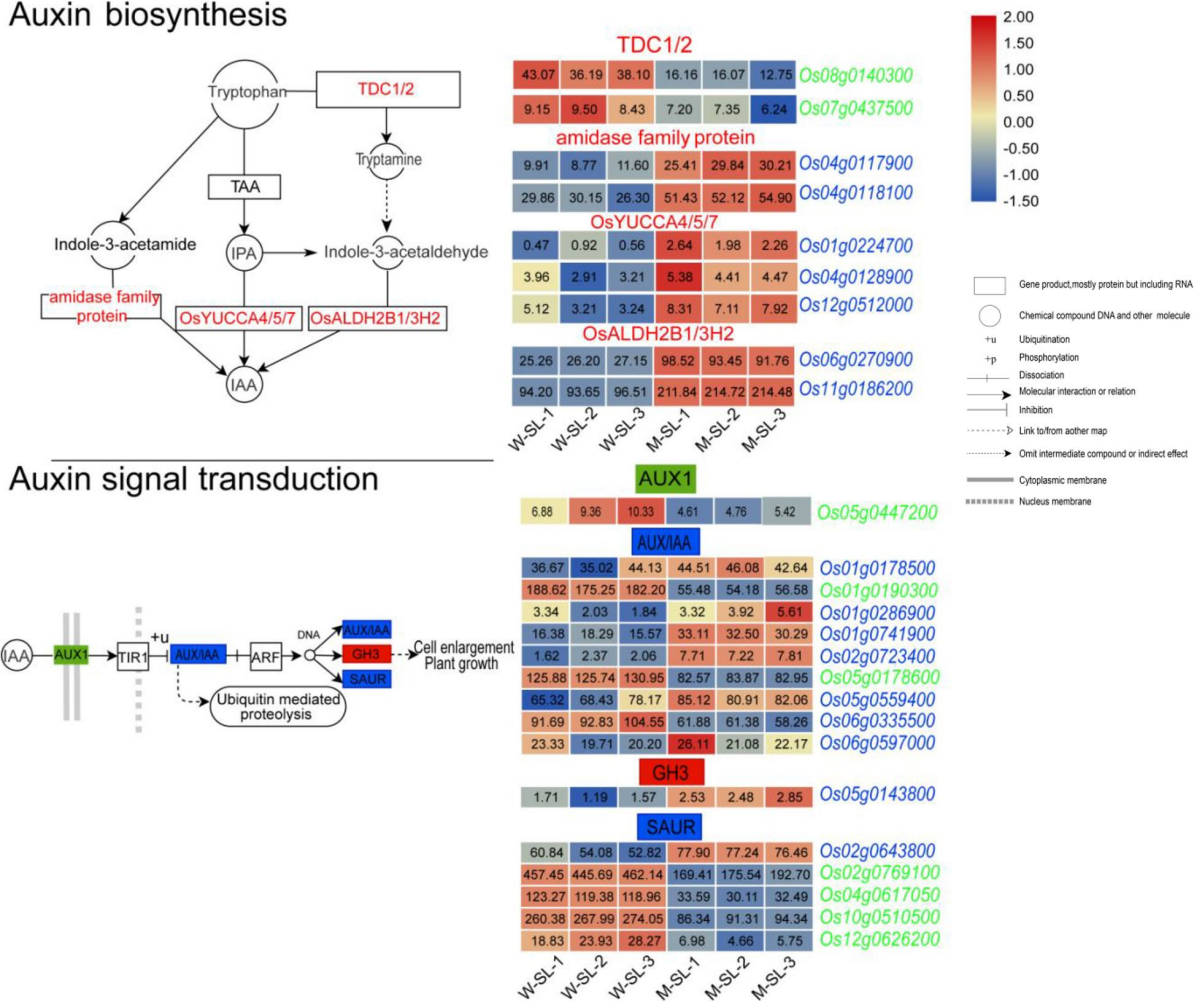
Identification of auxin-related genes in green and blue module. The red font represents identified auxin-related genes in the green or blue modules. The red rectangle represents upregulation of the expression of the corresponding genes, the green rectangle represents downregulation, and the blue rectangle represents both upregulation and downregulation of the expression of the corresponding genes. FPKM values were normalized as row scale

In rice, three main biosynthesis pathways from tryptophan to indole-3-acetic acid (IAA) have been researched [40]. In the current study, nine genes related to IAA biosynthesis and 16 genes related to auxin signal transduction were identified in the green or blue modules (Fig. 8). Three *YUCCA-like* genes were found in the blue module and were upregulation in the sterile lemmas of the mutant relative to the wild type (the fold change of *OsYUCCA4*, *OsYUCCA5* and *OsYUCCA7* was 1.83, 0.52 and 1.03, respectively). The *YUCCA* genes, which encodes flavin monooxygenase-like enzymes, and the rice enzyme OsYUCCA is thought to play an important role in IAA biosynthesis via the tryptophan-dependent pathway [41]. In addition, we identified nine *Aux/IAA* genes in green and blue modules (Fig. 8). Among them, *Os01g0190300* and *Os02g0723400* exhibited larger fold changes in the long, sterile lemma mutant (− 1.70 and 1.93, respectively). The Aux/IAA acts as a transcriptional repressor be triggered by the E3 ubiquitin ligase SCF^TIR1/AFB^, and thus, regulates auxin signaling [42]. Small auxin-up RNAs (SAURs) comprise a large multigene family and are rapidly activated as part of the primary auxin response in plants [43]. In the green module, four *SAUR* genes that were significantly downregulated in the mutant relative to the wild type were identified (fold change in the mutant was − 1.32, − 1.90, − 1.54 and − 2.01, respectively), among which *Os02g0769100* and *Os10g0510500* were among the HUB genes of the green module.

**Fig. 9:**
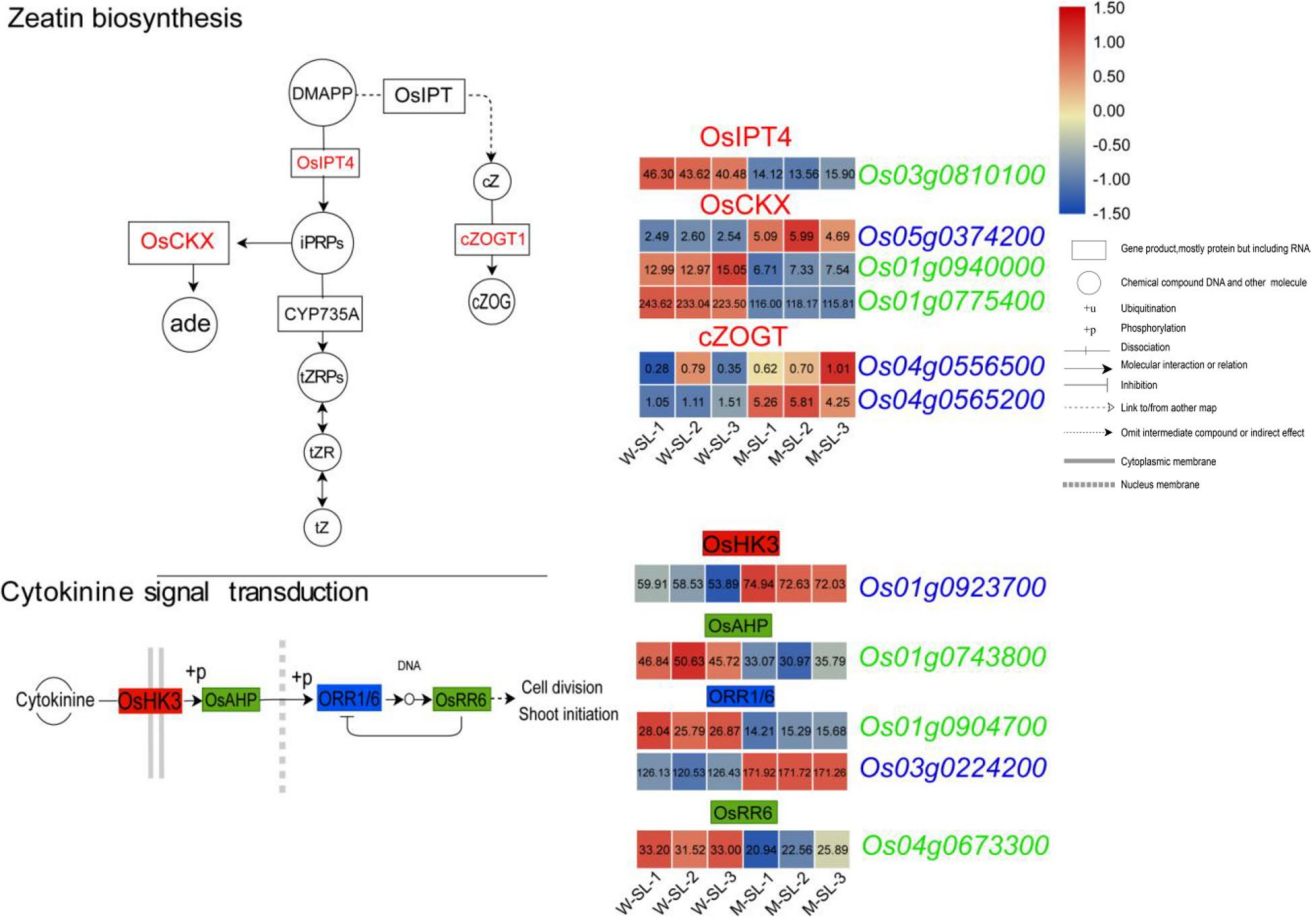
Identification of cytokinine-related genes in green and blue module. The red font represents identified auxin-related genes in the green or blue modules. The red rectangle represents upregulation of the expression of the corresponding genes, the green rectangle represents downregulation, and the blue rectangle represents both upregulation and downregulation of the expression of the corresponding genes. FPKM values were normalized as row scale

Cytokinin (CK) synthesis in rice begins with the synthesis of adenosine isoprene 5′ phosphate (iPRPs) from dimethylallyl diphosphate (DMAPP) and adenosine phosphate (ATP/ADP/AMP) catalyzed by isopentenyltransferases (IPTs). Ultimately, various cytokinins, such as *trans*-zeatin, *cis*-zeatin and dihydro-zeatin are synthesized. In the green and blue modules, we found six genes related to zeatin biosynthesis and five genes related to cytokinin signal transduction (Fig. 9). In the zeatin biosynthesis pathway, we noted the presence of an adenosine phosphate isopentenyltransferase gene, *OsIPT4*; these *IPT* genes catalyze the rate-limiting step of CK biosynthesis. The expression of *OsIPT4* was distinctly downregulated in the mutant (fold change is − 1.56). We also found a *cis*-zeatin *O*-glucosyltransferase gene, *Os04g0565200*, and the expression of this gene was significantly upregulation (fold change is 2.08). In rice, the cytokinin signal is transduced by the phosphotransfer mechanism [22]. A range of cytokinin receptors or response regulators are involved in CK signal transduction. We identified five CK signaling genes in the green and blue modules: one histidine kinase (HK) genes of the cytokinin receptor family *OsHK3*, one histidine-containing phosphotransfer gene, *Os01g0743800*, two B-type response regulator genes, *Os01g0904700*(*ORR1*) and *Os03g0224200*(*ORR6*) and one A-type response regulator gene, *Os04g0673300* (*OsRR6*). The expression levels of these genes were slightly altered (Fig. 9), indicating that the CK regulation was affected in the long, sterile lemmas of the mutant.

**Fig. 10:**
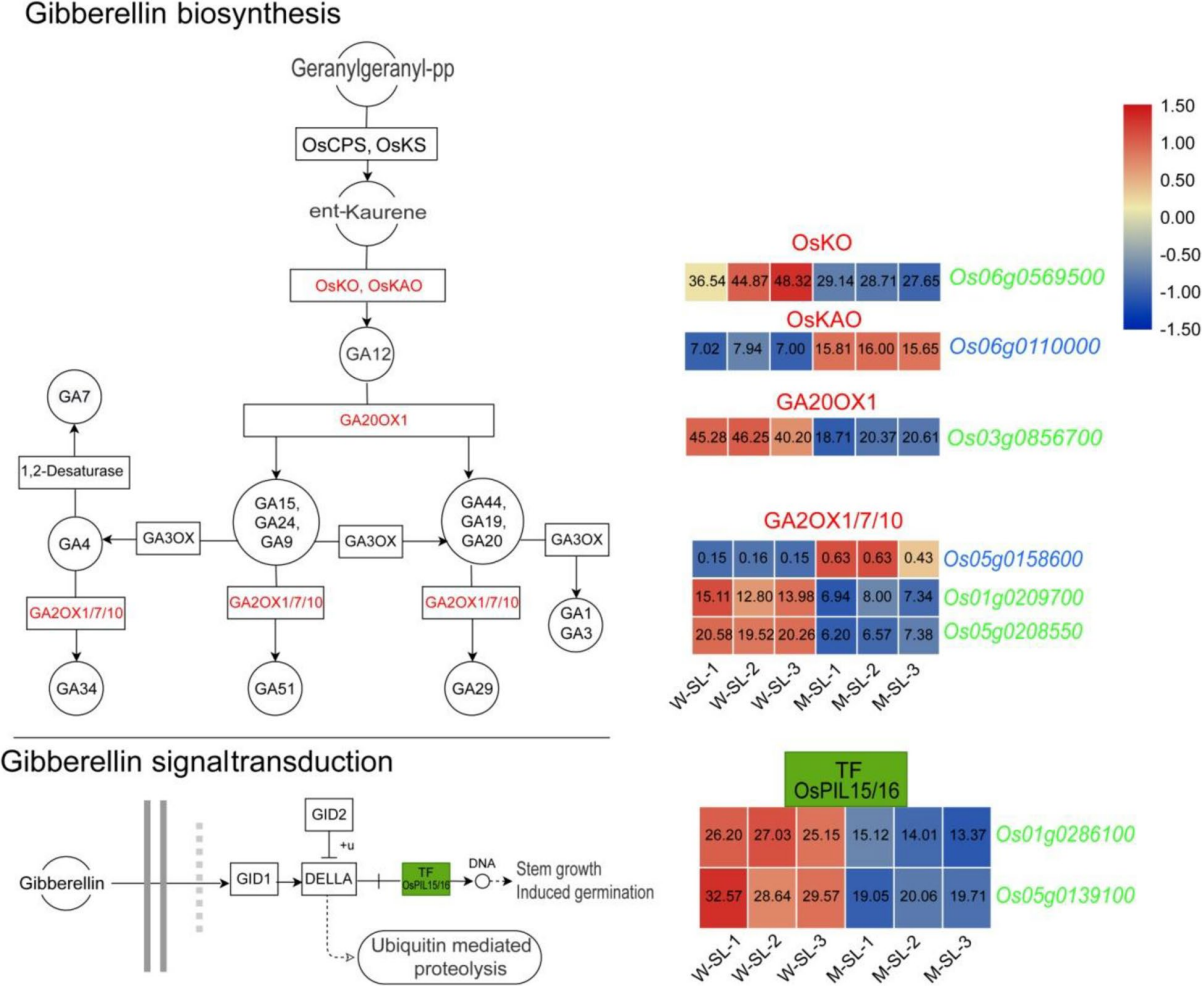
Identification of gibberellin-related genes in green and blue module. The red font represents identified auxin-related genes in the green or blue modules. The red rectangle represents upregulation of the expression of the corresponding genes, the green rectangle represents downregulation, and the blue rectangle represents both upregulation and downregulation of the expression of the corresponding genes. FPKM values were normalized as row scale

Gibberellins are biosynthesized in rice from geranylgeranyl diphosphate (GGDP) which is converted to *ent*-kaurene catalyzed by *ent*-copalyl diphosphate synthase (CPS) and *ent*-kaurene synthase (KS), *ent*-kaurene is subsequently converted to GA_12_ by two P450s monooxygenase enzymes, *ent*-kaurene oxidase (KO) and *ent*-kaurenoic acid oxidase (KAO). GA_12_ is converted by various GA oxidases into different gibberellin products [44]. In the green and blue modules, we found six genes related to gibberellin biosynthesis and two genes related to gibberellin signal transduction (Fig. 10). In the GA biosynthesis pathway, we identified the gene *OsGA20ox1* which coded for gibberellin (GA) 20-oxidase (GA20ox). As mentioned above, *OsGA20ox1* is the target gene of *OsLBD37* in the second type of regulatory network. *OsGA20ox1* was clearly downregulated in the long, sterile lemmas of the mutant (fold change was − 1.12). We also identified three gibberellin 2-oxidases (GA2oxs) genes, namely *GA2OX1*, GA20OX7 and *GA20OX10*, and the expression levels of these genes were altered in the sterile lemmas of the mutant (fold change is 1.87, − 0.89 and − 1.56, respectively). In the pathway of GA signal transduction, two phytochrome-interacting factor-like protein genes, *OsPIL15* and *OsPIL16* were identified in the green module. The transcript abundances of the two genes in the sterile lemmas of the mutant were slightly downregulated (Fig. 10). Previous studies had shown that *OsPIL15* [45, 46] and *OsPIL16* [47] were associated with grain size or secondary branch number of rice.

### Determination and analysis of phytohormone levels in the sterile lemmas of MT and WT

As mentioned above, the expression levels of genes encoding several key enzymes involved in phytohormone synthesis were significantly changed. To confirm whether the corresponding hormone levels in the sterile lemmas of the mutant were changed, we determined phytohormone concentrations in the sterile lemmas of the mutant and wild type using the method of ultra-high-performance liquid chromatography–tandem mass spectrometry (UHPLC−MS/MS).

**Fig. 11:**
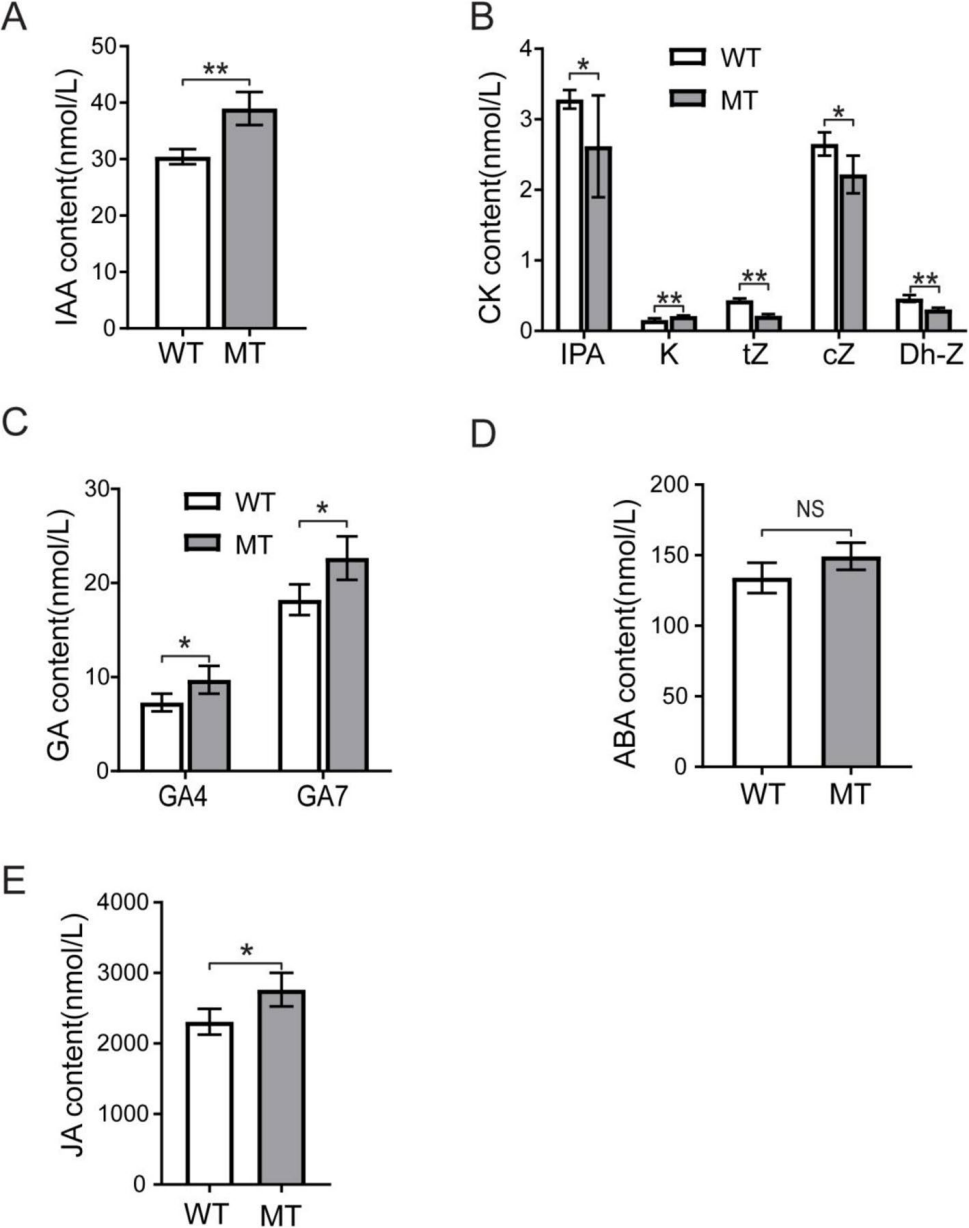
Analysis of hormone concentrations differences in sterile lemmas between mutant (MT) and wild-type (WT) rice. **a** Concentrations differences of IAA in sterile lemmas between mutant and wild-type rice. **b** Concentrations differences of CK in sterile lemmas between mutant and wild-type rice. **c** Concentrations differences of GA in sterile lemmas between mutant and wild-type rice. **d** Concentrations differences of ABA in sterile lemmas between mutant and wild-type rice. **e** Concentrations differences of JA in sterile lemmas between mutant and wild-type rice. Analysis was carried out by the Student’s t-test. NS indicates not significant, * indicates a *P* < 0.05, ** *P* < 0.01, error line indicate mean ± SD. IAA: indole-3-acetic acid, CK: cytokinin, GA: gibberellin, ABA: abscisic acid, JA: jasmonic acid

The concentration of the auxin indole-3-acetic acid (IAA) in the mutant sterile lemmas at the booting stage was upregulated compare with the wild type, which might be caused by the increased expression of *YUCCA* and *ALDH* genes (Fig. 11a). In the sterile lemma, the concentrations of the isopentenyl adenosine (IPA), *trans*-zeatin (tZ), *cis*-zeatin (cZ) and dihydro-zeatin (Dh-Z) were significantly lower in the MT than in the WT. On the contrary, the kinetin (K) concentrations of increased significantly in the MT. We speculate that this finding might be related to the changes in expression level of the isopentenyl transferase gene *OsIPT4* and the cytokinin dehydrogenase gene *OsCKX* (Fig. 11b). The concentrations of four major bioactive GAs, namely GA_1_, GA_3_, GA_4_, and GA_7_, were determined. In neither mutant nor wild type were GA_1_ or GA_3_ detected in the sterile lemmas. However, GA_4_ and GA_7_ were detected and the concentrations in the MT sterile lemmas were significantly higher than in the WT (Fig. 11c).

We also determined the concentrations of abscisic acid (ABA), jasmonic acid (JA) and brassinolactone (BR) in the sterile lemmas of MT and WT (Fig. 11d, e). The results showed that the ABA concentration was not significantly different between MT and WT. The concentration of JA in the sterile lemmas was higher than those of other hormones, and the concentration of JA in the mutant was increased significantly higher than in the WT. While BR was not detected in sterile lemmas of either the mutant or the wild type.

### *G1* is involved in phytohormone regulation in sterile lemmas of rice

Previous studies had suggested that *G1* functions as a mediator or co-repressor in controlling the expression of genes that have a direct impact on the growth of sterile lemmas [10]. In the current study, a large number of genes associated with sterile lemmas development was identified, and many of these genes are involved in the biosynthesis or signal transduction of phytohormones.

Among the five modules related to sterile lemma development, the proportion of transcription factors in the green module was the highest, and the expression of the most genes were downregulated in the sterile lemmas of the mutant. The first type of regulatory network is centered on the green module transcription factors, of which *OsLBD37* and *OSH1*, two vital transcription factors which control flower development of rice, have attracted our attention. In order to determine whether there is a closer regulatory relationship, we constructed a regulatory network of transcription factors in the green module using the GENIE3 algorithm. Four transcription factors, namely OSH1, OsLBD37, HL6 and GRAS, are located at the center of the network and regulate multiple downstream genes. Among these target genes are three phytohormone genes, *OsGA20ox1*, *OsIAA15, P450* (Fig. S13). The results of quantitative reverse transcription PCR (qRT-PCR) showed that the expression levels of the seven genes were downregulated in the mutant, and the expression levels of each gene were consistent with the transcriptome sequencing data, with positive correlations between qRT-PCR and RNA-Seq results (Fig. S14).

**Fig. 12:**
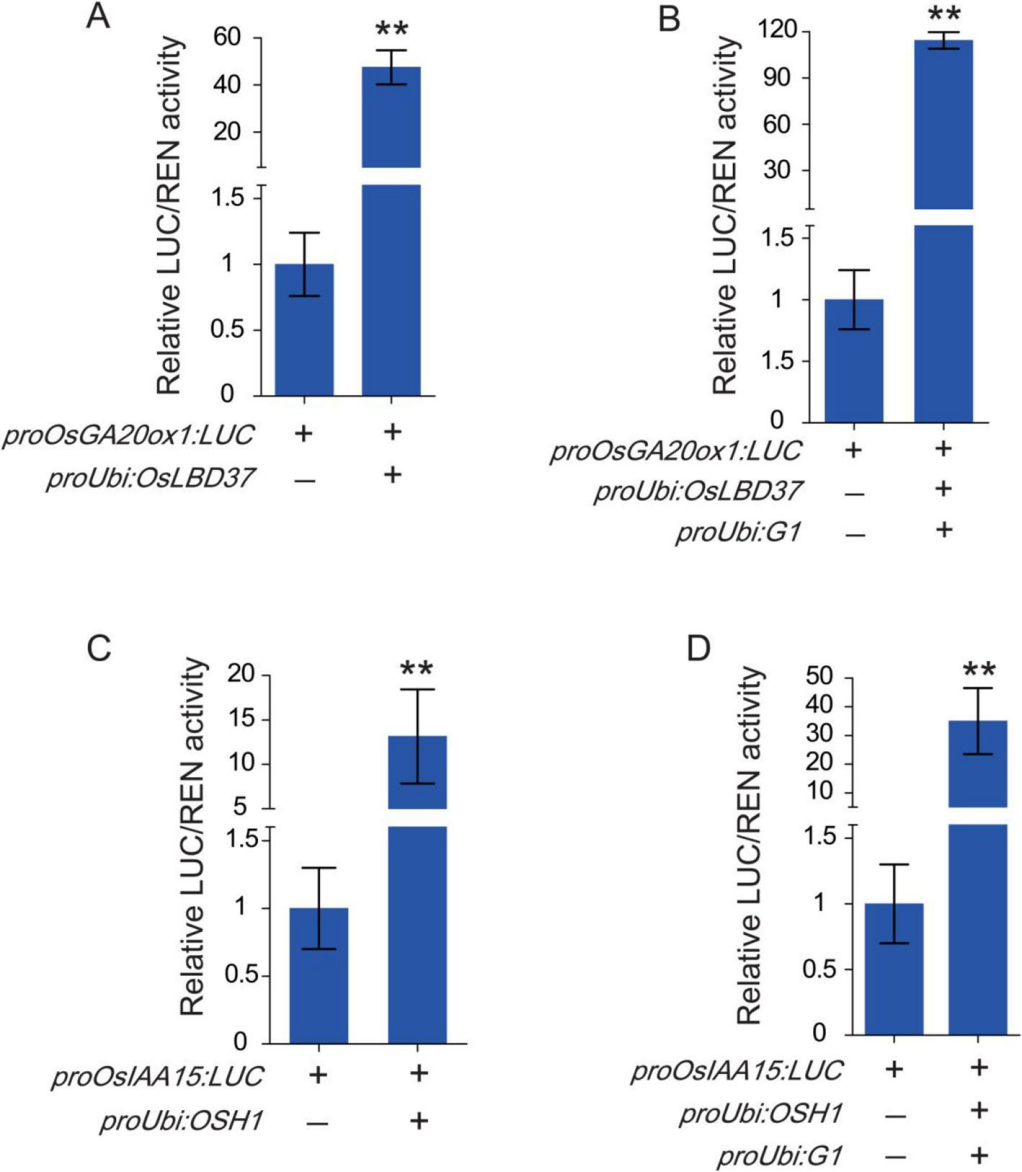
In vivo validation of the *G1-OsLBD37-OsGA20ox1* and *G1-OSH1-OsIAA15* regulation in co-transfection assays. Reporter plasmids containing the firefly luciferase (LUC) gene and renilla luciferase (REN) activities were co-transfected into rice protoplasts. The plus symbol (+) represents that the plasmid is contained in the protoplast and the minus symbol (−) represents that it is not contained. Error lines represents mean ± SD, Two-tailed Student’s *t*-test was performed to determine the significance of the transcription factor effects. ** represents *P* < 0.01. **a** Co-transfection assays of proOsGA20ox1:LUC and proUBI:OsLBD37. **b** Co-transfection assays of *proOsGA20ox1:LUC*, *proUBI:OsLBD37* and *proUBI:G1*. **c** Co-transfection assays of *proOsIAA15:LUC* and *proUBI:OSH1*. **d** Co-transfection assays of *Pro OsIAA15:LUC*, *proUBI:OSH1* and *proUBI:G1*

From the regulatory network analysis, we speculated that *OsLBD37* positively regulates *OsGA20ox1* and *OSH1* positively regulates *OsIAA15*. To verify the above predictions and confirm whether *G1* is involved in the regulation of these two groups of genes, we designed and performed co-transfection assays in rice protoplasts. Four groups of experiments were designed. Group A: *proOsGA20ox1:LUC* and *proUBI:OsLBD37*; Group B: *proOsGA20ox1:LUC*, *proUBI:OsLBD37* and *proUBI:G1*; Group C: *proOsIAA15:LUC* and *proUBI:OSH1*; Group D: *Pro OsIAA15:LUC*, *proUBI:OSH1* and *proUBI:G1*. Each group was co-transfected into rice protoplasts. The single *proOsGA20ox1:LUC* plasmid or the *proOsIAA15:LUC* plasmid was transfected into rice protoplasts as the control. When OsLBD37 or OSH1 proteins were present, activity of the luciferase reporter gene was significantly increased compared with the control. These results confirmed that *OsLBD37* positively regulates *OsGA20ox1* and *OSH1* positively regulates *OsIAA15* (Fig. 12a, c). When the *proUBI:G1* plasmid was added to groups A or C, luciferase activity further increased compared with the control (Fig. 12b, d). In general, both *G1-OsLBD37-OsGA20ox1* and *G1-OSH1-OsIAA15* exhibited cascade regulatory patterns, and *G1* was involved in the regulation of gibberellins and auxin.

## Discussion

To date, there are many genes which can affect sterile lemma development in rice have been cloned. We have summarized 12 genes that involved in different stages of rice flower development even plant development [8], Thus, in addition to morphological changes in the sterile lemma, functional mutations in these genes resulted in a variety of phenotypic alterations. However, only the mutants of *G1* showed specific changes in the sterile lemma, although, as found in the present study, it also have caused an increased secondary branch number, which may be an indirect effect. Therefore, *G1* mutant was selected as the basic material for studying sterile lemma development.

Yoshida et al. first identified the *G1* gene in the long, sterile lemma mutant of a variety of *japonica* rice in 2009. Since then, many alleles of *G1/ ELONGATEDEMPTY GLUME (ELE)* have been reported [9–11]. These studies revealed a role for *G1/ELE* in inhibiting the transition from sterile lemma to lemma. However, the mechanism of how *G1* regulates downstream genes, thereby controlling sterile lemma development, remains unclear. In the current study, to analyze the regulatory mechanism of sterile lemma development by *G1*, we performed a comprehensive analysis of transcriptome dynamics during sterile lemma development in the mutant with the long, sterile lemma phenotype (caused by the mutant allele of *G1/ELE*) and its wild type.

Using WGCNA, we obtained five modules related to sterile lemma development. The genes in each module had the same expression pattern at the five developmental stages of the sterile lemmas. WGCNA not only allowed us to find the gene sets related to the sterile lemma phenotype, but also divided these gene sets into specific modules that were either upregulated or downregulated at early or late stage of sterile lemma development. WGCNA greatly reduced the scale of the search for potential key genes of sterile lemma development and provided a certain directionality.

Although gene sets with similar expression patterns could be obtained by WGCNA, the regulatory relationships among various genes could not be described. Thus, we adopted the machine-learning approach GENIE3 algorithm to infer the regulatory relationships between various transcription factors and their target genes. Several regulatory relationships confirmed by previous studies were found in the regulatory network. For example, *OsNAC2* downregulated the IAA signaling-related gene *OsARF25* [34, 35]; the *KNOX* family gene *OSH1* regulates *OSH15* [28] and *OSH1* regulates the GA20 oxidase gene *OsGA20ox1* [48]. Therefore, the regulatory networks predicted by the GENIE3 algorithm have considerable reference value, and some of the regulatory relationships with strong correlations are worthy of further investigation.

Preliminary statistics of RNA-Seq data suggest that there were still thousands of differentially expressed genes (DEGs) at each stage of sterile lemma development. We identified 259 DEGs and performed GO enrichment analysis that overlaps in five samples. Compare with GO enrichment analysis of 420 target genes (predicted by the GENIE3 algorithm), both the top 20 GO terms with significant enrichment were related to ‘biological processes’. But the top 20 GO terms of the 259 DEGs mainly related to ‘response’, while the top 20 GO terms of the 420 target genes are related to ‘regulation’. This distinction implies that the 259 DEGs belongs to some kind of ‘dependent variable’, while the 420 target genes belongs to some kind of ‘independent variable’ in the regulatory network of sterile lemma development. Therefore, we infer that most of the 420 target genes are upstream regulatory genes compared to the 259 DEGs in the regulatory network. In the top 20 GO terms with significant enrichment of 420 target genes, we analyzed target genes in four GO terms: ‘flower development’, ‘plant organ formation’, ‘reproductive shoot system development’ and ‘regulation of developmental process’. Eight genes appeared many times in the four GO terms and were related to synthesis or signal transduction of gibberellins and auxin (Table. S8).

It is worth noting that *YUCCA6* can be directly regulated by the lateral spikelet gene *LF1*, the protein product of which can directly bind to the promoter of the gene *OSH1*, activate the ectopic expression of *OSH1*, and initiate the formation of the lateral flower meristem [49]. In first type of regulatory network predicted by this study, *YUCCA6* can be regulated by the basic helix-loop-helix transcription factor *RAI1* [50]. Compared with other target genes, the expression levels of *YUCCA6* was low in five samples of WT and MT. However, the expression level of *YUCCA6* was slightly upregulated in the S4 and SL samples of the mutant (Fig. S15). These results indicate that both *G1* and *LF1* can affect the expression of *YUCCA6*, thereby maintaining the normal phenotype of the spikelets.

*FZP* (*FRIZZY PANICLE*) was another target gene among the eight genes. Previous studies [32, 51–53] had shown that the sterile lemmas of the *fzp* mutant produced supernumerary rudimentary glumes or degraded sterile lemmas. In addition, *FZP*-overexpression induced a homologous change of the sterile lemma to the lemma. RNA in situ hybridization analysis showed that *FZP* was expressed not only at the meristem of rudimentary glumes, but also at the meristem of sterile lemmas, lemmas and paleas, although *FZP* was not expressed in the meristem of the inner floret organs [53]. In the present study, RNA-Seq data showed that *FZP* in the wild type was slightly expressed at stages S1 and S2 of young panicle development, but was not expressed at stages S3 or S4 nor in sterile lemmas at the booting stage (Fig. S15). These results indicate that *FZP* was expressed during meristem formation of sterile lemmas, but expressed at extremely low levels in differentiated sterile lemmas. However, the expression levels of *G1* in the wild type were higher during stages S1 − S4. Thus, we speculate that the mild downregulation of *FZP* expression at stage S2 of the mutant was not directly caused by the *G1* mutation. In the second regulatory network, *FZP* was predicted to be a target gene of *OsARF25* and *OsARF4*, so that the expression of *FZP* might be influenced by auxin at an early stage of young panicle development.

On the other hand, Gibberellin 20-oxidase gene *OsGA20ox1* was identified in the first type of regulatory network, but it was also a crucial gene that was associated with flower development in rice. Previous studies had shown that higher *OsGA20ox1* transcript abundance levels increased expression of the GA catabolism genes *GA2oxs*, reduced GA_1_ and GA_3_ accumulation, and increased cytokinin activity, thereby increasing grain number and grain yield in rice [48]. In the present study, the downregulation of *OsGA20ox1* transcription levels in the sterile lemmas of the mutant caused a series of effects contrary to previous studies. In the sterile lemmas of mutant at the booting stage, the transcript levels of two *GA2oxs* genes were significantly decreased; the levels of most cytokinin intermediates were decreased and the concentration of gibberellins (GA_4_ and GA_7_) were increased significantly, with the secondary branch number decreasing significantly. *OsLBD37* is a class II type LBD (LATERAL ORGAN BOUNDARIES DOMAIN) protein: LBD proteins are plant-specific transcription factors which are involved in many biological processes of plant development [29, 54, 55]. In the current study, *OsLBD37* and *OsGA20ox1* were predicted to have a strong regulatory relationship. *OsLBD37* is also associated with development of the panicle in rice, and the panicle length of *OsLBD37*-overexpression lines was more than 3 cm longer than that of control plants, while the grain number per panicle were almost doubled [29]. In the present study, transient expression regulation assay in rice protoplasts at seedling stage confirmed that the OsLBD37 protein enhanced the expression of *OsGA20ox1* and G1 protein further raised the transcript levels of *OsGA20ox1*. These findings suggest that *G1* may affect the phenotype of the sterile lemmas and secondary branch number through participating in *OsLBD37* and *OsGA20ox1* regulation.

Knotted1-like homeobox (KNOX) transcription factors play crucial roles in establishment and maintenance of the shoot apical meristem (SAM). *KNOX* genes are considered to inhibit cell differentiation in the SAM by decreasing and increasing the levels of GAs and CKs, respectively [28, 56]. In the present study, the expression of *KNOX* gene *OSH1* was downregulated significantly in the sterile lemmas of the mutant. The concentrations of GAs and CKs in the sterile lemma of the mutant increasing and decreasing respectively. This result is consistent with that from previous studies, so we speculate that the downregulation of *KNOX* genes reduced the inhibitory effect on cell differentiation and induced the overgrowth of the sterile lemmas of the mutant.

A typical model for the auxin signal transduction pathway is that high concentration of auxins can directly stimulate the interaction between Aux/IAA proteins and SCF^TIR1^ E3 ubiquitin-ligase complexes, resulting in the degradation of Aux/IAA proteins [57–59], so that ARF transcription factors are released from inhibition and can regulate the expression of auxin-responsive target genes [60]. In the regulatory network of the green module, the *Aux/IAA* gene *OsIAA15* is the target gene of *OSH1.* Both RNA-Seq data and qRT-PCR data showed that expression of *OsIAA15* was downregulated. Meanwhile, transient expression regulation assay in rice protoplasts confirmed that the OSH1 protein enhanced the expression of *OsIAA15*, and G1 protein can further increased the transcript abundance of *OsIAA15*. These results indicate that *G1* may regulates *OSH1* expression to maintain low auxin levels in the sterile lemmas, thereby inhibiting overgrowth of the sterile lemmas.

Taken together, *G1* affects the expression of downstream genes (including various structural genes for enzymes and signaling genes) by regulating a range of critical transcription factors. The stable expression of these downstream genes maintains the homeostasis of phytohormones in the sterile lemmas of rice. This hormone balancing starts from the cellular differentiation of sterile lemmas at the early stages and retains stability until the whole spikelet matures. The determination of hormone levels in the sterile lemmas of WT and MT supports the model that lower IAA, lower GA, and higher CK are required to maintain the normal phenotype of the sterile lemmas. In conclusion, these studies provide a comprehensive guide to facilitate further study of the molecular mechanism of sterile lemma development.

### Experimental procedures

#### Selection of plant materials and RNA sample collection

The mutant with the phenotype of long, sterile lemmas was identified from a ^60^Coγ-ray-radiation-induced population derived from *indica* rice cv. Huanghuazhan from Guangdong Academy of Agricultural Sciences, Guangzhou, Guangdong, China. In previous work, we determined that the phenotype of the long, sterile lemmas in the mutant is caused by the mutant allele of *G1/ELE*, with the mutant being named *g1–7*. The mutant and its wild type were planted in a greenhouse of the Rice Research Institute, Fujian Academy of Agricultural Sciences in Fuzhou, China, in 2019. We sampled young panicles at four stages and sterile lemma at booting stage (Table 1) from *g1–7* and the wild type for tissue used for RNA-Seq, qRT-PCR and hormone concentration measurement (Table 1). After sampling, the tissues were snap-frozen in liquid nitrogen and stored at –70 °C until RNA extraction. Three biological replicates were used for each of the genotypes and sampling points.

#### Analysis of phenotype

The spikelets at the flowering stage were dissected and observed under stereomicroscope. Photos to record the shape of flower organs of the mutant and wild type.

Sterile lemma length, panicle length, effective panicle number, primary branch number, secondary branch number, grains per panicle, seed setting rate and 1000-grain weight of *g1–7* mutant and the wild type were measured at the stage of yellow ripeness. Analysis of variance and drawing of boxplots was conducted using the “ggsignif” and “ggplot2” package of the R software, and Student^’^s *t*-test was used to determine whether the mean values of the two genotypes were significantly different.

#### RNA extraction, library construction and sequencing

The total RNA of each of above listed samples (Table 1) was isolated using the TRIzol Kit (Promega, Madison, WI, USA) following by the manufacturer’s instructions. Then the total RNA was treated with RNase-free DNase I (Takara Bio, Kusatsu, Shiga, Japan) for 30 min at 37 °C to remove residual DNA. RNA quality was verified using a 2100 Bioanalyzer (Agilent Technologies, Santa Clara, CA, USA) and was also checked by R electrophoresis on RNase free agarose gels.

Next, Poly (A) mRNA was isolated using oligo-dT beads (QIAGEN, Pudong, Shanghai, China). All mRNA was broken into short fragments by adding fragmentation buffer. First-strand cDNA was generated using random hexamer-primed reverse transcription, followed by the synthesis of the second-strand cDNA using RNase H and DNA polymerase I. The cDNA fragments were purified using a QIAquick PCR Extraction Kit (QIAGEN, Pudong, Shanghai, China). These purified fragments were then washed with EB buffer for end reparation poly (A) addition and ligated to sequencing adapters. Following agarose gel electrophoresis and extraction of cDNA from gels, the cDNA fragments were purified and enriched by PCR to construct the final cDNA library.

The cDNA library was sequenced on the Illumina sequencing platform (Illumina HiSeq™ 2000) using the paired-end technology by Gene Denovo Co. (Guangzhou, China). An in-house Perl program was written to select clean reads by removing low quality sequences (there were more than 50% bases with quality lower than 20 in one sequence), reads with more than 5% N bases (bases unknown) and reads containing adaptor sequences.

#### Data processing of RNA-Seq experiments

The clean data obtained after filtering were compared with the reference genome of the species (https://www.ncbi.nlm.nih.gov/genome/10?genome_assembly_id=256442). HTSeq (a tool designed for short sequences alignment) was used to calculate the read count value of each gene as the original expression amount of the gene. The expression was normalized using FPKM (Fragments Per Kilo bases Per Million Fragments). For paired-end sequencing, each fragment will have two reads, FPKM only calculates two reads to compare the number of fragments in the same transcript. Gene with FPKM> 1 were used as the next step in data analysis.

#### Differentially expressed genes (DEGs) and principal component analysis (PCA)

After the expression level of each gene was calculated, differential expression analysis was conducted using edgeR [61]. The false discovery rate (FDR) was used to determine the threshold of the *p*-value in multiple tests, and for the analysis, a threshold of the FDR ≤ 0.01 and |log2 (fold change)| > 1 were used to judge the significance of the gene expression differences. Using the expression matrix of 35,666 genes in 30 samples as input data, principal component analysis and 2D scatter plots were performed using the “FactoMineR”, “ggplot2” and “ggrepel” packages of the R software.

#### Weighted gene co-expression network analysis (WGCNA)

With the expression matrix of 35,666 genes in 30 samples as input data, the co-expression network was established by the WGCNA program package [14] based on the R language. The samples with more than 10% deletions, the genes with variance = 0, and the genes with more than 10% deletions were filtered out.

The “blockwiseModules” function was used to convert the expression matrix into adjacency matrix, and then the adjacency matrix was converted into a topology matrix. The specific parameters are set as follows: power = 18; minModuleSize = 20,150; TOMType = “unsigned”; deepSplit = 2; minModuleSize = 30; mergeCutHeight = 0.25.

According to the soft threshold, each gene is assigned to a module of corresponding color; if a gene does not belong to any module, it is presented as ‘grey’. Output expression heatmap and bar chart of eigengenes were determined for all modules.

Function commands “moduleTraitCor” and “moduleTraitPvalue” were used to calculate the correlation coefficient and *P*-value between ME (Module Eigengene) and the sample. Then the output heatmap of the correlation coefficients was obtained. The “edge” file and “datKME” file for each module. Selected the genes that top 20 Kme values (the expression correlation between a gene and the eigengene in one module) in each module. Any hypothetical proteins were removed, the top 300 weights (correlation between genes) from the “Edge” file were extracted, then the diagram of the network was drawn with Cytoscape version 3.7.1 [62], and the genes in the top 10 degree values were regarded as HUB genes.

#### Identification of transcription factors and prediction of gene regulatory networks

Amino acids sequences of gene products in the five modules were submitted to the iTAK website (http://itak.feilab.net/cgi-bin/itak/index.cgi) [63], then all transcription factors were identified. Using FPKM of 35,666 genes in 30 samples as expression matrix, all transcription factors in the five module served as regulatory genes, with the GENIE3 (Gene Network Inference with Ensemble of Trees) package in R [64] being used for regulatory network inference. The top 500 links were extracted and the diagram of network regulation was drawn with Cytoscape version 3.7.1. In the green module, the FPKM of 1318 genes in 30 samples was used as the expression matrix and the top 100 links were extracted to draw the diagram of network regulation.

#### Gene ontology (GO) enrichment analysis

TBtools version 1.09 [65] was used for GO enrichment analysis of different gene sets. The go-basic.obo file used for enrichment analysis was downloaded from the GO official website (http://purl.obolibrary.org/obo/go/go-basic.obo). Background files of rice (*Oryza sativa japonica*) used for GO enrichment analysis were extracted from the eggnog-Mapper website (http://eggnog-mapper.embl.de/). TBtools was used to draw the histogram of enrichment results. A histogram showed the top 20 GO terms of significant enrichment.

#### Identification of plant hormone related genes

The rice genome sequence was downloaded (*Oryza sativa japonica*) from EnsemblPlant website (http://plants.ensembl.org/Oryza_sativa/Info/Index). DNA sequences were converted to amino acid sequences using TBtools software. Amino acid sequences were submitted to the KEGG website (https://www.kegg.jp), and 10,198 IDs were obtained which had KEGG annotation numbers. The genes associated with biosynthesis or signal transduction of auxin, cytokinines, and gibberellins in green and blue modules were identified. According to the map of hormone synthesis and signaling transduction on the KEGG website [66], a schematic diagram was drawn and labelled with the genes identified, using Adobe illustrator software (CS5, Version 15.0.0).

#### Extraction and determination of phytohormones in the sterile lemmas

The extraction of phytohormones in sterile lemma was achieved as described in previous studies [67]. Ultra-high-performance liquid chromatography–tandem mass spectrometry (UHPLC-MS/MS) was used to determine the content of phytohormones.

The UHPLC separation was carried out using an EXIONLC System, equipped with a Waters ACQUITY UPLC CSH C18 column (150 × 2.1 mm, 1.7 μm, Waters). The mobile phase A was 0.01% formic acid in water, and the mobile phase B was 0.01% formic acid in acetonitrile. The column temperature was set at 50 °C. The auto-sampler temperature was set at 4 °C and the injection volume was 5 μl.

A SCIEX 6500 QTRAP+ triple quadrupole mass spectrometer, equipped with an IonDrive Turbo V electrospray ionization (ESI) interface, was applied for assay development. Typical ion source parameters were: Curtain Gas = 40 psi, IonSpray Voltage = ±4500 V, temperature = 475 °C, Ion Source Gas 1 = 30 psi, Ion Source Gas 2 = 30 psi.

#### Quantitative reverse transcription-PCR analysis

Total RNA was reverse-transcribed using ReverTra Ace qPCR RT Master Mix with gDNA Remover kit (Toyobo, japan). 1:20 cDNA was used as the template in qPCR. FastStart Universal SYBR Green Master (ROX) (Roche, USA) was used to set up the PCR reactions, which were run and analyzed on a LightCycler 480II machine (Roche, https://www.roche.com.cn/). The results were quantified and normalized relative to the endogenous control gene (*UBQ5*) using the comparative 2^-ΔΔCT^method [68]. Primer sequences to target genes associated with hormones are listed in Supplemental Table S9.

#### Transient expression regulatory assay

The promoter of *OsGA20ox1* and *OsIAA15* were amplified and ligated into the pGreenII 0800-LUC vector [69]. The coding sequence (CDS) of *OsLBD37*, *OSH1* and *G1* were cloned into the pRTVcHA vector. The effector and reporter constructs were co-transformed into rice protoplasts as reported previously [70]. The luciferase and renilla luciferase activities were measured using the Dual Luciferase Assay Kit (Vazyme, China). The analysis was executed by the CLARIOstar microplate reader (BMG Labtech, Germany), and the REN values were used for normalization. Details regarding the primers used for this assay are listed in Table S10.

## Supplementary Information

**Additional file1.**

**Additional file 2.**

**Additional file 3.**

**Additional file 4.**

**Additional file 5.**

**Additional file 6.**

**Additional file 7.**

**Additional file 8.**

**Additional file 9.**

**Additional file 10.**

**Additional file 11.**

**Additional file 12.**

**Additional file 13.**

**Additional file 14.**

**Additional file 15.**

**Additional file 16.**

## Abbreviations

WT: wild-type
MT: mutant
RNA-seq: RNA-Sequencing
qRT-PCR: Quantitative real-time polymerase chain reaction
SRA: Sequence Read Archive
DEGs: Differentially expressed genes
PCA: Principal component analysis.
WGCNA: Weighted Correlation Network Analysis
GENIE3: Gene Network Inference with Ensemble of trees
GO: Gene Ontology
KEGG: Kyoto Encyclopedia of Genes and Genomes
K_ME_: Eigengene-based connectivity
FPKM: Fragments per kilobase of transcript per million mapped reads
IAA: 3-Indolebutyric acid
CK: Cytokinin
cZ: cis-Zeatin
tZ: trans-Zeatin
IPA: N6-isopentenyladenosine
Dh-Z: DL-Dihydrozeatin
K: Kinetin
GA4: Gibberellin A4
GA7: Gibberellin A7
ABA: Abscisic acid
JA: Jasmonic acid
BR: Brassinolide
SAM: Shoot apical meristem
