# Supplementary figures and images for: Analysis of co-expression and gene regulatory networks associated with sterile lemma development in rice
Source: BMC Plant Biol. 2023 Jan 6;23:11. doi: 10.1186/s12870-022-04012-x (PMC9817312; doi:10.1186/s12870-022-04012-x)

**A**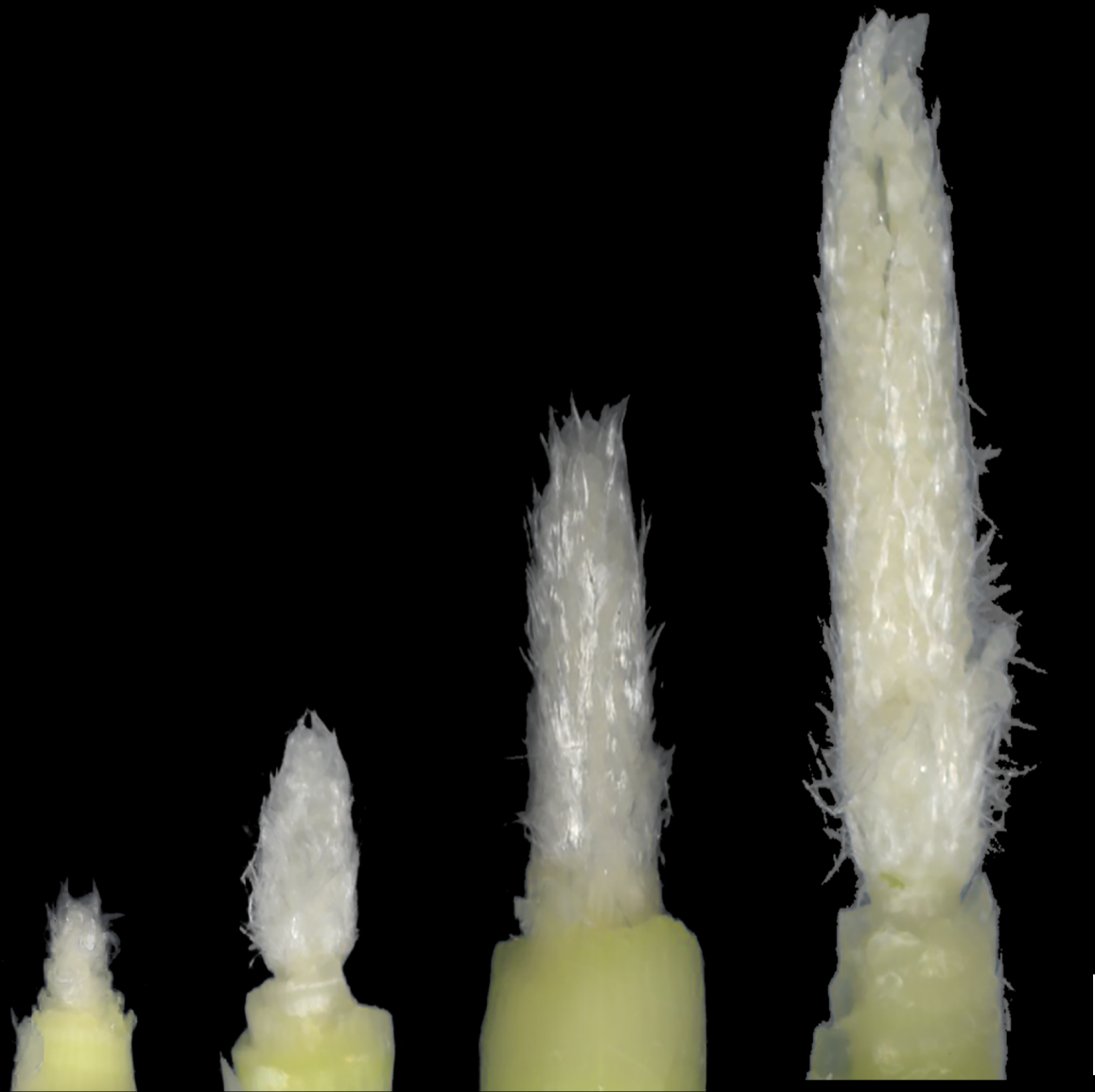**S1****S2****S3****S4****B**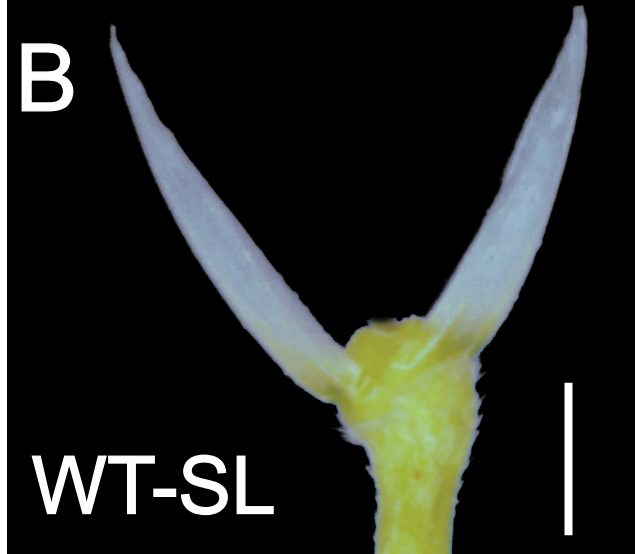**WT-SL****C**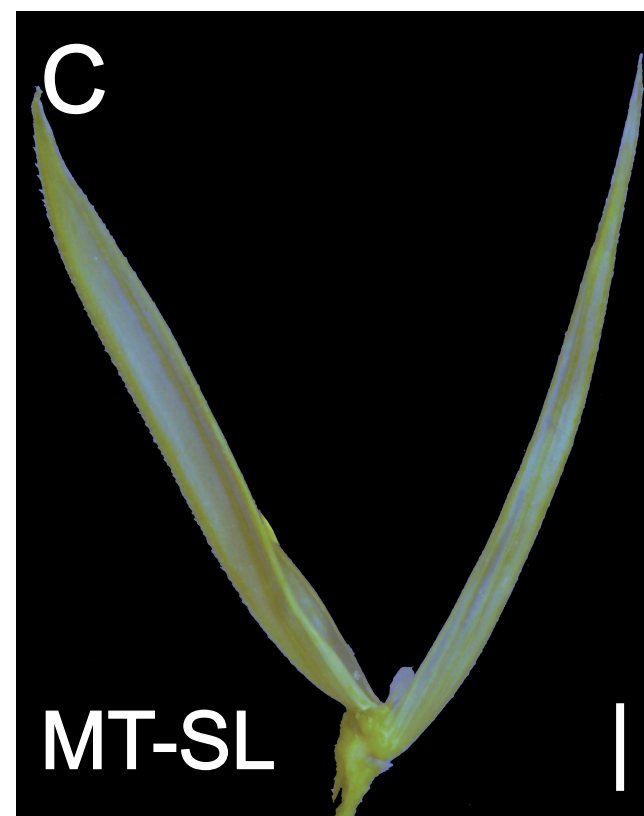**MT-SL**

**Fig. S1.** Sampling point of wild type and mutant. scale bar=1 mm.

Supplement: Supplementary file 1 — Additional file1. [file 12870_2022_4012_MOESM1_ESM.pdf]

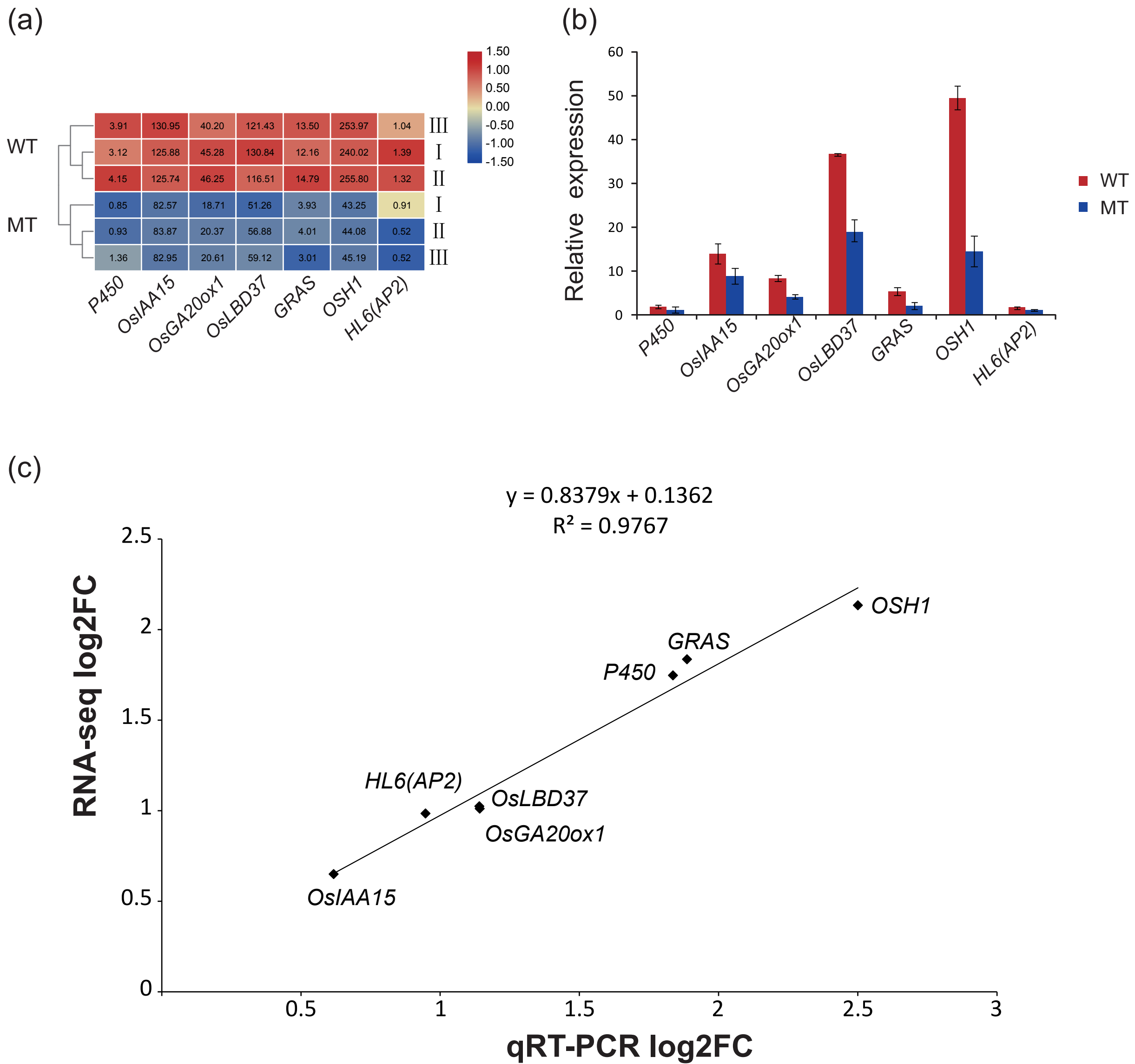

**Fig. S14.** Verification of the expression of seven key genes in the green module.

Supplement: Supplementary file 6 — Additional file 6. [file 12870_2022_4012_MOESM6_ESM.pdf]

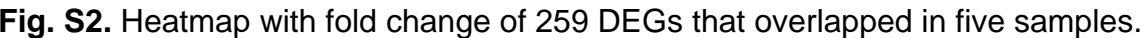

**Fig. S2.** Heatmap with fold change of 259 DEGs that overlapped in five samples.

Supplement: Supplementary file 8 — Additional file 8. [file 12870_2022_4012_MOESM8_ESM.pdf]

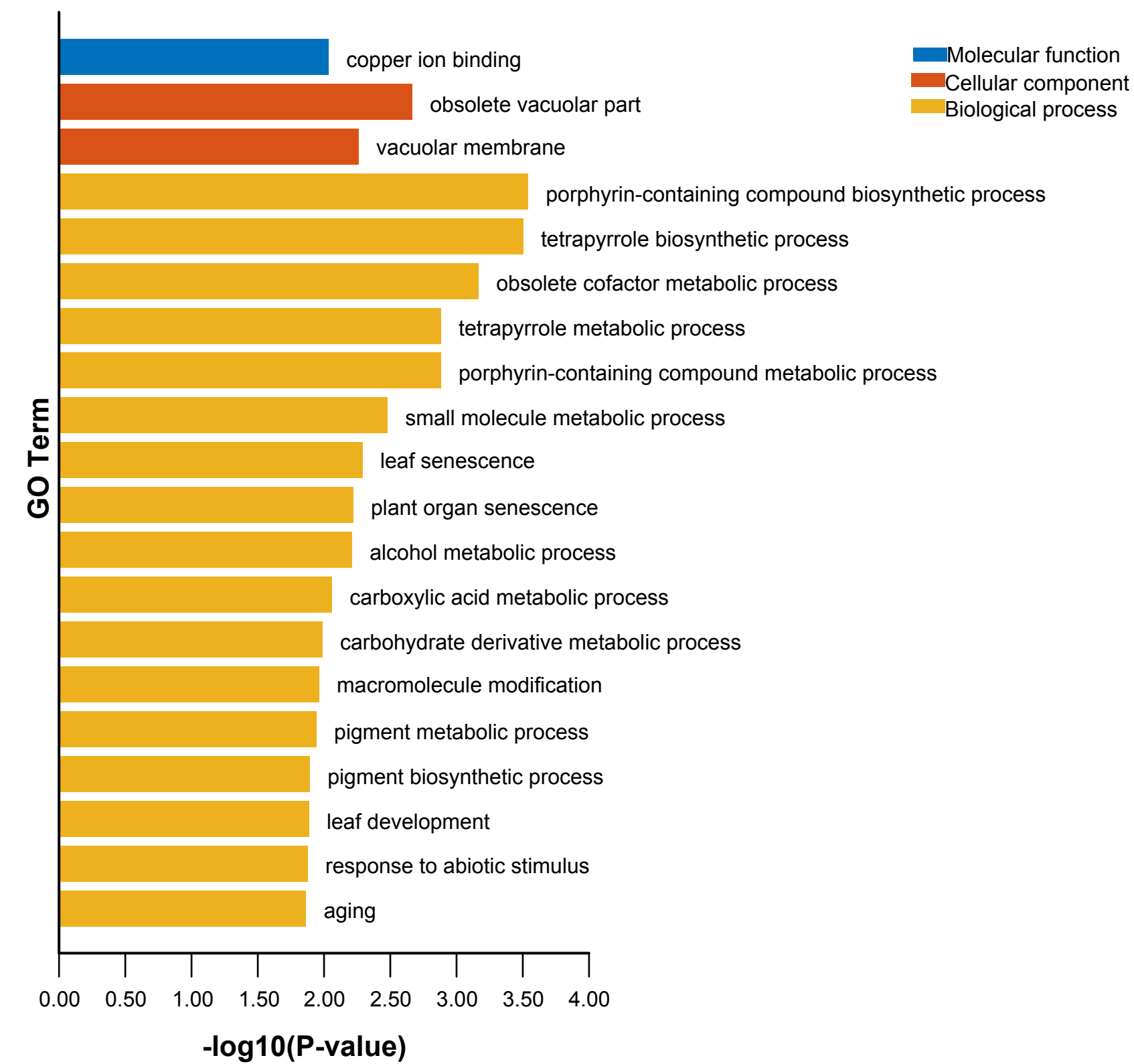

**Fig. S4.** GO enrichment and co-expression network of the black module

Supplement: Supplementary file 10 — Additional file 10. [file 12870_2022_4012_MOESM10_ESM.pdf]

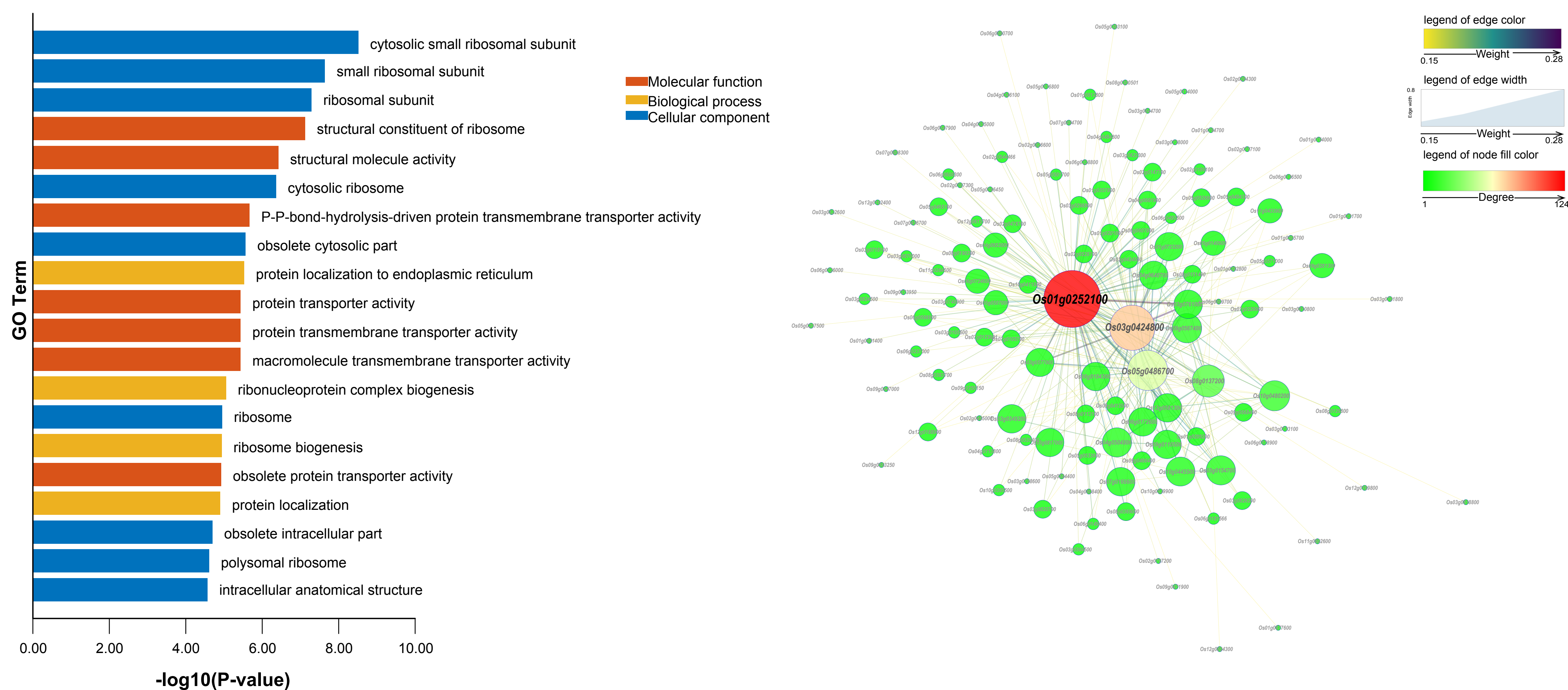

**Fig. S6.** GO enrichment and co-expression network of the yellow module

Supplement: Supplementary file 12 — Additional file 12. [file 12870_2022_4012_MOESM12_ESM.pdf]

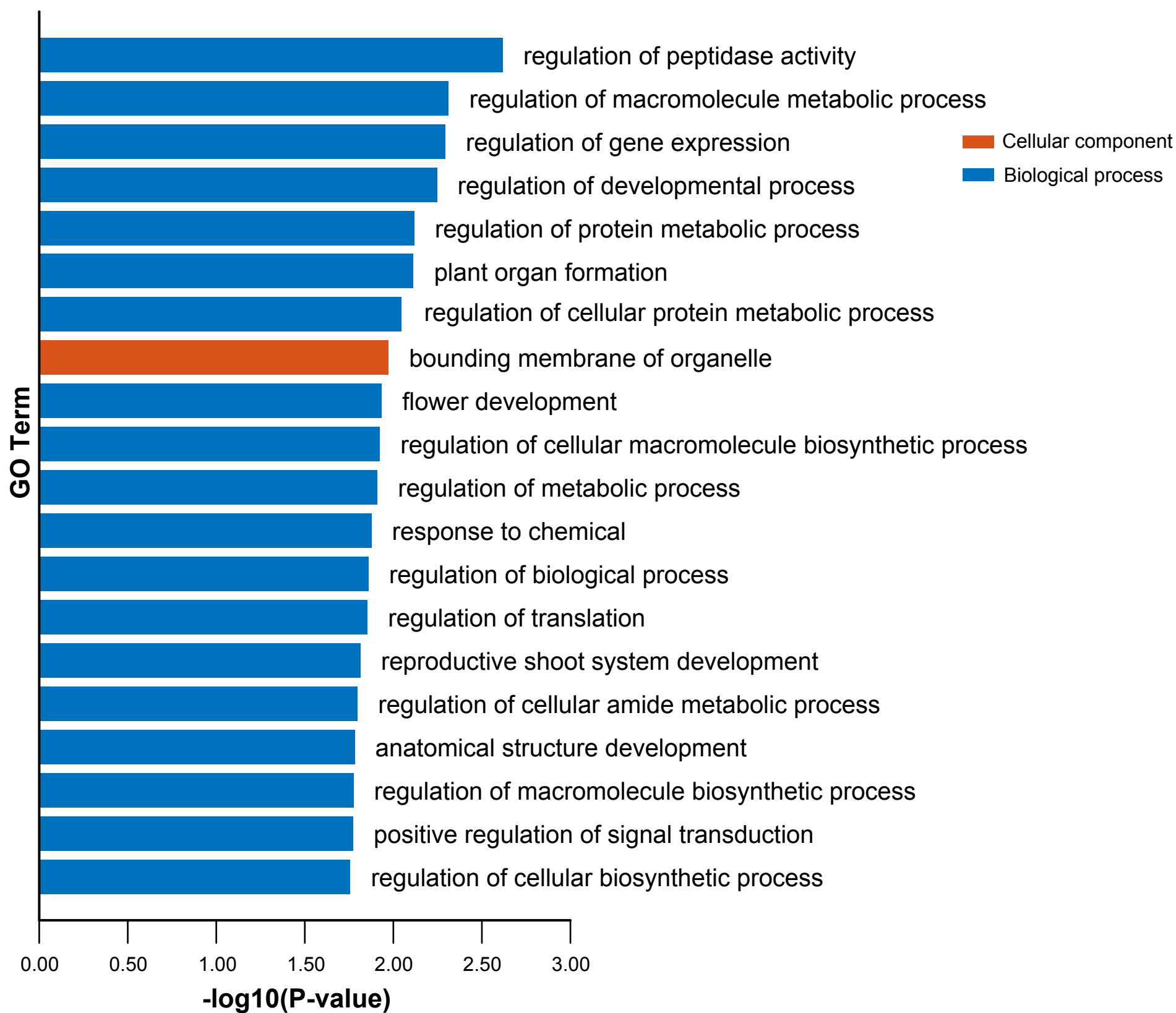

**Fig. S9.** GO enrichment analysis of 420 target genes.

Supplement: Supplementary file 15 — Additional file 15. [file 12870_2022_4012_MOESM15_ESM.pdf]
