# Supplementary material for: Analysis of co-expression and gene regulatory networks associated with sterile lemma development in rice
Source: BMC Plant Biol. 2023 Jan 6;23:11. doi: 10.1186/s12870-022-04012-x (PMC9817312; doi:10.1186/s12870-022-04012-x)
Supplement: Supplementary file 2 — Additional file 2. [file 12870_2022_4012_MOESM2_ESM.pdf]

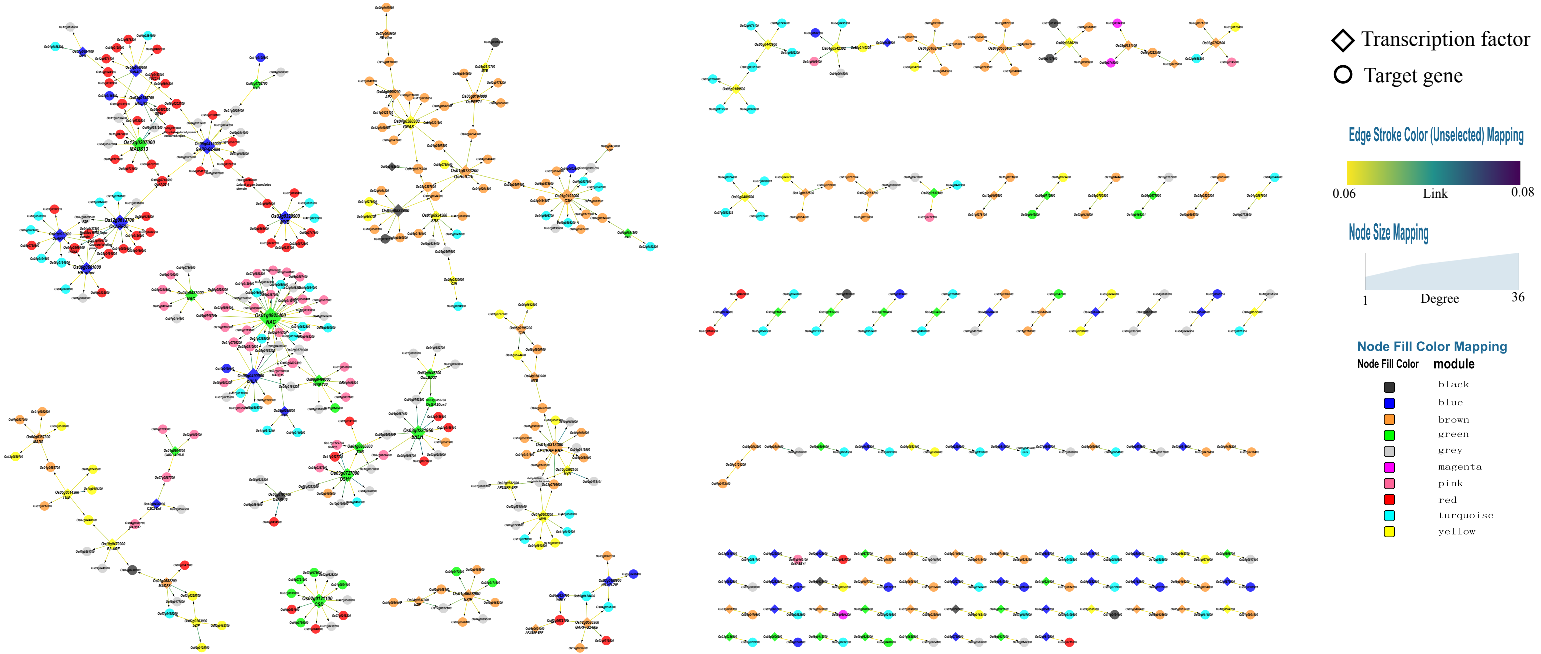


**Fig. S10.** The regulatory network of top 500 links.
