# Supplementary material for: Analysis of co-expression and gene regulatory networks associated with sterile lemma development in rice
Source: BMC Plant Biol. 2023 Jan 6;23:11. doi: 10.1186/s12870-022-04012-x (PMC9817312; doi:10.1186/s12870-022-04012-x)
Supplement: Supplementary file 4 — Additional file 4. [file 12870_2022_4012_MOESM4_ESM.pdf]

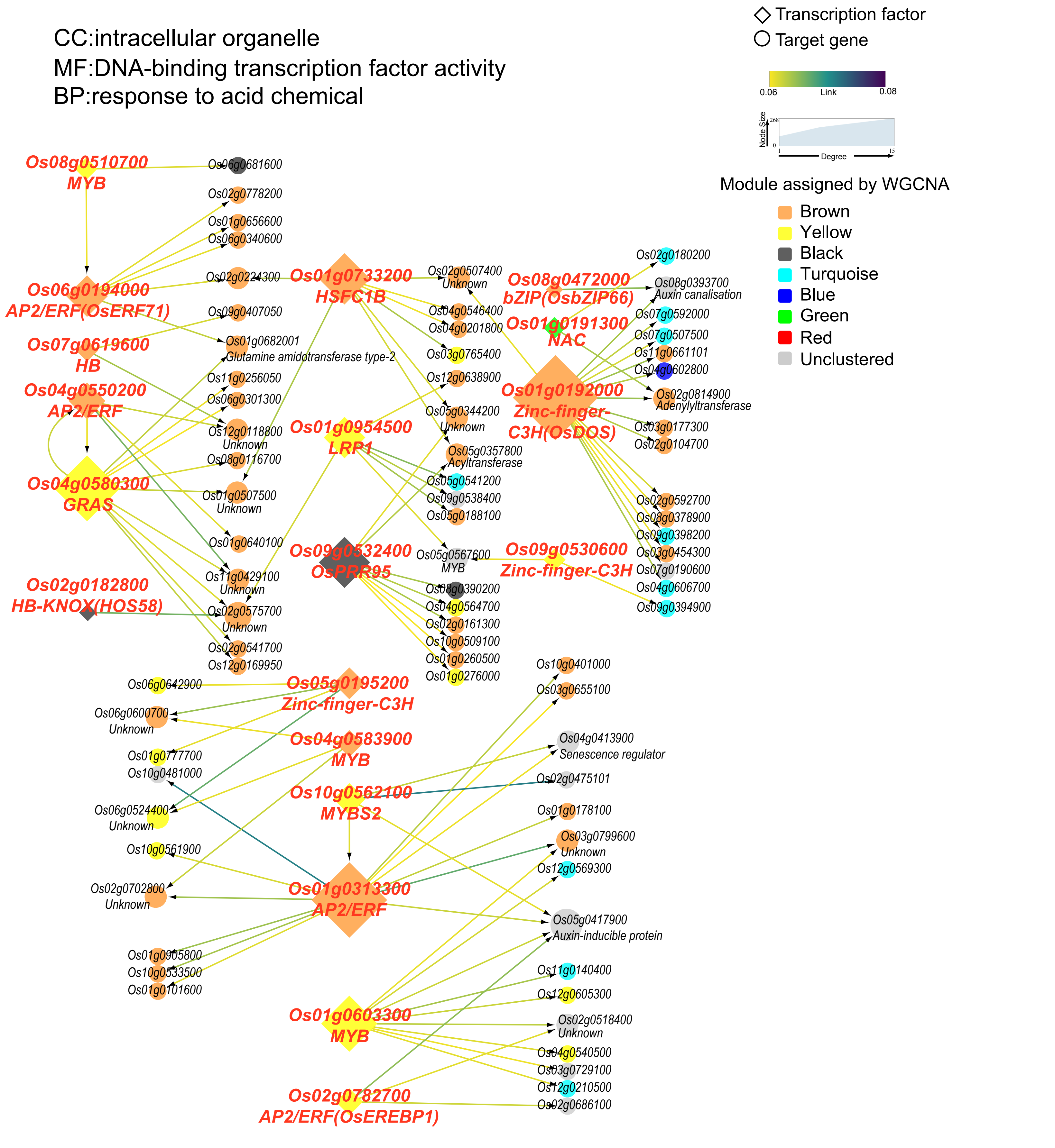

Fig. S12. The third type of regulatory network. The annotations in the upper left corner are the GO terms that minimize the *P*-value in the enrichment analysis of all genes in the network; CC: cell components; MF: Molecular function; BP: Biological process. Transcription factors are annotated with symbols or family names; descriptions of target genes regulated by more than two transcription factors are annotated.
