# Supplementary material for: Analysis of co-expression and gene regulatory networks associated with sterile lemma development in rice
Source: BMC Plant Biol. 2023 Jan 6;23:11. doi: 10.1186/s12870-022-04012-x (PMC9817312; doi:10.1186/s12870-022-04012-x)
Supplement: Supplementary file 7 — Additional file 7. [file 12870_2022_4012_MOESM7_ESM.pdf]

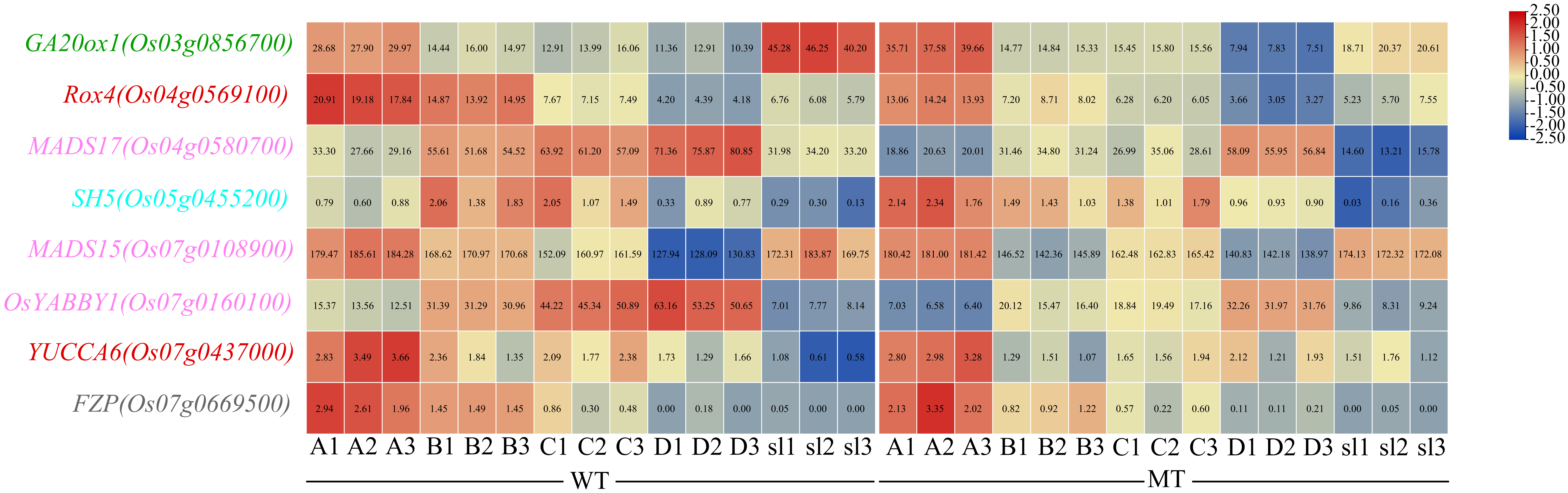

Fig. S15. The transcriptional levels of eight crucial genes that are known to regulate flower development in rice.
