# Supplementary material for: Analysis of co-expression and gene regulatory networks associated with sterile lemma development in rice
Source: BMC Plant Biol. 2023 Jan 6;23:11. doi: 10.1186/s12870-022-04012-x (PMC9817312; doi:10.1186/s12870-022-04012-x)
Supplement: Supplementary file 9 — Additional file 9. [file 12870_2022_4012_MOESM9_ESM.pdf]

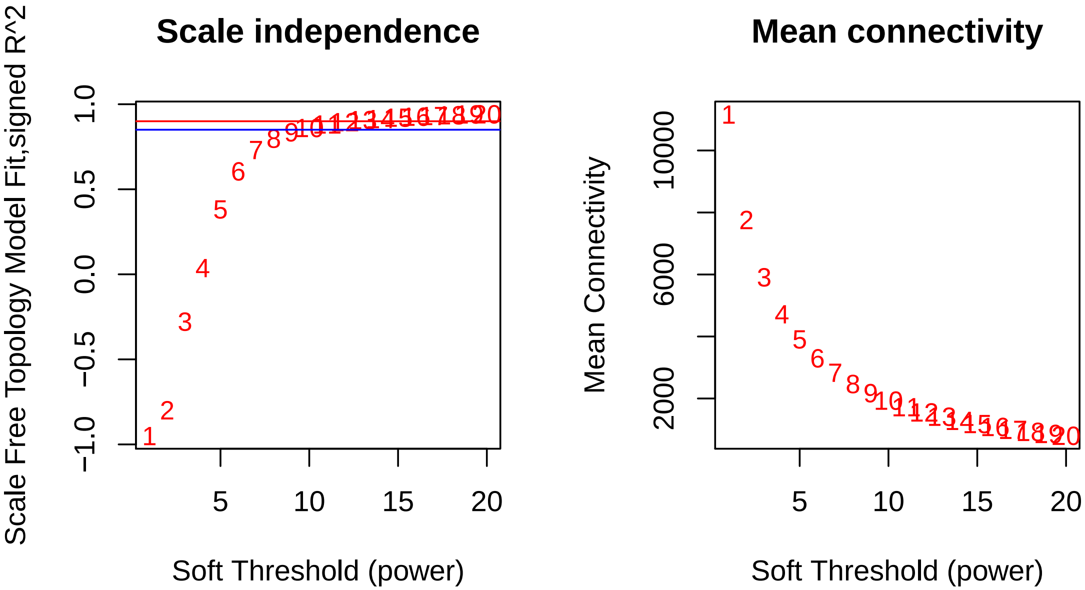

Fig. S3. R^2 and mean connectivity calculation of soft threshold.
