# Supplementary material for: Analysis of co-expression and gene regulatory networks associated with sterile lemma development in rice
Source: BMC Plant Biol. 2023 Jan 6;23:11. doi: 10.1186/s12870-022-04012-x (PMC9817312; doi:10.1186/s12870-022-04012-x)
Supplement: Supplementary file 16 — Additional file 16. [file 12870_2022_4012_MOESM16_ESM.doc]

[**Table**](javascript:;) **S1** Description of 26 genes associated with hormone response

| Gene ID | Description |
| --- | --- |
| *Os01g0785400* | GH3 auxin-responsive promoter |
| *Os01g0221100* | GH3 auxin-responsive promoter |
| *Os01g0762500* | Seed storage protein |
| *Os01g0148000* | Putative zinc finger in N-recognin (UBR box) |
| *Os01g0176700* | Myb-like DNA-binding domain |
| *Os01g0682001* | Glutamine amidotransferases class-II(GLT1) |
| *Os01g0588200* | membrane that allows diffusion of small hydrophilic molecules. The channel adopts an open conformation at low or zero membrane potential and a closed conformation at potentials above 30-40 mV. The open state has a weak anion selectivity whereas the closed state is cation- selective (By similarity)(VDAC3) |
| *Os02g0664100* | UDP-glucose:Glycoprotein Glucosyltransferase |
| *Os02g0244700* | phosphoenolpyruvate carboxylase |
| *Os02g0685200* | Myb-like DNA-binding domain |
| *Os03g0192700* | Inositol-3-phosphate synthase |
| *Os03g0744675* | Beige/BEACH domain |
| *Os04g0475600* | Belongs to the iron ascorbate-dependent oxidoreductase family |
| *Os05g0392050* | E3 ubiquitin-protein ligase KEG |
| *Os05g0530500* | Non-specific serine threonine protein kinase |
| *Os05g0536200* | Eukaryotic porin(VDAC1) |
| *Os05g0457200* | Protein phosphatase 2C |
| *Os06g0255200* | Chromatin structure-remodeling complex protein |
| *Os06g0132500* | hydrolase, alpha beta fold family |
| *Os08g0561500* | EamA-like transporter family |
| *Os10g0505900* | Low-temperature-induced 65 kDa |
| *Os10g0575000* | bHLH-MYC and R2R3-MYB transcription factors N-terminal |
| *Os11g0184900* | No apical meristem (NAM) protein(NAC5) |
| *Os11g0454000* | Belongs to the plant dehydrin family(DHN1) |
| *Os11g0454200* | Belongs to the plant dehydrin family(DHN1) |
| *Os11g0454300* | Belongs to the plant dehydrin family(DHN1) |

**Table S2** Identification of HUB gene in black module

| Gene ID | Description | Degree | Kme | number of significantly enriched GO terms | Research level in rice | Gene symbol |
| --- | --- | --- | --- | --- | --- | --- |
|
| *Os01g0762500* | Seed storage protein Glutelin subunit mRNA | 28 | 0.98 | 0 | Function research | *GLUA1* |
| *Os07g0188800* | Similar to Methyl malonate-semialdehyde dehydrogenase | 26 | 0.97 | 18 | Function research | *OsALDH6B2* |
| *Os02g0730000* | Similar to Mitochondrial aldehyde dehydrogenase | 23 | 0.97 | 1 | Function research | *OsALDH2B5* |
| *Os07g0421866* | Similar to tetratricopeptide repeat (TPR)-containing protein | 23 | 0.95 | 2 | Protein structure prediction | — |
| *Os01g0785400* | GH3 auxin-responsive promoter family protein | 20 | 0.95 | 6 | Function research | *OsGH3-1* |
| *Os06g0199100* | Helix-turn-helix, AraC type, subdomain 2 domain containing protein | 15 | 0.98 | 5 | Protein structure prediction | — |
| *Os01g0880200* | Glycosyl transferase, family 8 protein | 15 | 0.95 | 3 | Protein structure prediction | — |
| *Os03g0351300* | Similar to beta-amylase | 14 | 0.92 | 2 | Protein structure prediction | — |
| *Os06g0258900* | Ketose-bisphosphate aldolase class-II family protein | 14 | 0.96 | 19 | Transcriptional expression | — |
| *Os03g0310400* | Crotonase core domain containing protein | 14 | 0.92 | 7 | Protein structure prediction | — |

The number of significantly enriched GO annotations reflects that there is more known information about this gene. The same as below.

**Table S3** Identification of HUB gene in brown module

| Gene ID | Description | Degree | Kme | number of significantly enriched GO terms | Research level | Gene symbol |
| --- | --- | --- | --- | --- | --- | --- |
|
| *Os05g0563100* | Similar to GTP binding / GTPase | 63 | 0.990 | 14 | Protein structure prediction | — |
| *Os08g0154600* | DNA Topoisomerase 1, Modulation of auxin-regulated root development and gravitropis | 63 | 0.990 | 30 | Protein structure prediction | — |
| *Os03g0210400* | RNA-processing protein, HAT helix domain containing protein | 55 | 0.989 | 49 | Protein structure prediction | — |
| *Os09g0121050* | Similar to SMC6 protein | 54 | 0.988 | 44 | Protein structure prediction | — |
| *Os03g0790600* | Glutamate carboxypeptidase, Hormone homeostasis, Regulation of developmental processe | 53 | 0.961 | 24 | Function research | *PLA3/GO* |
| *Os05g0506200* | KOW domain containing protein | 47 | 0.984 | 40 | Protein structure prediction | — |
| *Os09g0279000* | Similar to r-interacting factor1 | 23 | 0.984 | 7 | Protein structure prediction | — |
| *Os10g0559700* | Similar to Agenet domain containing protein, expressed | 6 | 0.962 | 0 | Protein structure prediction | — |
| *Os05g0446600* | ROS1D Similar to DNA glycosylase/lyase 701 | 6 | 0.959 | 0 | Protein structure prediction | — |
| *Os06g0505302* | Similar to SWEETIE (SWEETIE); binding | 6 | 0.971 | 18 | Protein structure prediction | — |

| Gene ID | Description | Degree | Kme | Number of significantly enriched GO terms | Research level | Gene symbol |
| --- | --- | --- | --- | --- | --- | --- |
|
| *Os01g0252100* | Similar to Glycogen synthase kinase-3 homolog MsK-3 | 124 | 0.981 | 10 | Transcriptional expression | — |
| *Os03g0424800* | Similar to 40S ribosomal protein S19-3 | 75 | 0.986 | 55 | Transcriptional expression | *RPS19A* |
| *Os05g0486700* | Ribosomal protein L24e domain containing protein | 54 | 0.986 | 55 | Transcriptional expression | — |
| *Os08g0137200* | Actin/actin-like family protein | 21 | 0.985 | 27 | Transcriptional expression | — |
| *Os10g0480200* | Homeobox-leucine zipper protein HOX9 | 13 | 0.987 | 6 | Function research | *HOX9* |
| *Os09g0567400* | Histidine phosphotransfer protein, Cytokinin signaling and stress respons | 10 | 0.968 | 20 | Function research | *OsAHP2*/*OHP2* |
| *Os04g0504800* | Similar to Poly(A)-binding protein | 9 | 0.985 | 13 | Transcriptional expression | — |
| *Os03g0154700* | Similar to 40S ribosomal protein S9 | 9 | 0.987 | 60 | Transcriptional expression | — |
| *Os10g0445500* | HR-like lesion-inducer family protein | 8 | 0.975 | 0 | Transcriptional expression | — |
| *Os03g0761000* | SWIB/MDM2 domain containing protein | 6 | 0.933 | 27 | Transcriptional expression | — |

**Table S4** Identification of HUB gene in yellow module

**Table S5** Identification of HUB gene in blue module

| Gene ID | Description | Degree | Kme | number of significantly enriched GO terms | Research level | Gene symbol |
| --- | --- | --- | --- | --- | --- | --- |
|
| *Os08g0502700* | Pyridoxal phosphate-dependent transferase, major region, subdomain 1 domain containing protein | 35 | 0.999 | 24 | Transcriptional expression | — |
| *Os09g0425900* | Similar to senescence-associated protein DH | 31 | 0.999 | 0 | Protein structure prediction | — |
| *Os08g0389700* | Conserved hypothetical protein | 30 | 0.999 | 0 | Protein structure prediction | — |
| *Os03g0207400* | Similar to Protein phosphatase 2C-like | 29 | 0.999 | 3 | Transcriptional expression | — |
| *Os02g0326700* | Peptidase S54, rhomboid domain containing protein | 26 | 0.999 | 4 | Protein structure prediction | — |
| *Os04g0460600* | NAC transcription factor | 24 | 0.999 | 0 | Function research | *OsNAC2* |
| *Os11g0182900* | Ankyrin repeat domain containing protein | 19 | 0.986 | 0 | Protein structure prediction | — |
| *Os04g0543900* | Glutamate dehydrogenase 2, mitochondrial | 19 | 0.999 | 31 | Function research | *GDH2* |
| *Os02g0101500* | Peroxisomal hydroxypyruvate reductase, NADH- dependent HPR, Photorespiratory metabolis | 19 | 0.988 | 3 | Transcriptional expression | — |
| *Os02g0777800* | Protein kinase, catalytic domain domain containing protein | 18 | 0.983 | 3 | Protein structure prediction | — |

**Table S6** Identification of HUB gene in green module

| Gene ID | Description | Degree | Kme | number of significantly enriched GO terms | Research level | Gene symbol |
| --- | --- | --- | --- | --- | --- | --- |
|
| *Os07g0191650* | Similar to F-box domain containing protein, expressed | 39 | 0.997 | 5 | Protein structure prediction | — |
| *Os08g0409100* | Trehalose-6-phosphate phosphatase | 38 | 0.997 | 1 | Transcriptional expression | — |
| *Os10g0510500* | Auxin responsive SAUR protein family protein | 37 | 0.998 | 6 | Protein structure prediction | — |
| *Os03g0170900* | Sucrose transporter | 33 | 0.996 | 25 | Function research | *OsSUT1* |
| *Os02g0300700* | Similar to Eukaryotic translation initiation factor 1A (EIF-1A) (EIF-4C) | 32 | 0.996 | 0 | Transcriptional expression | *eif-1a-OF1* |
| *Os03g0103400* | GRAS transcription factor domain containing protein | 29 | 0.997 | 5 | Protein structure prediction | *—* |
| *Os05g0500500* | Heat shock protein Hsp20 domain containing protein | 23 | 0.997 | 1 | Transcriptional expression | *HSP22.3* |
| *Os02g0301100* | Bidirectional sugar transporter SWEET4 | 21 | 0.996 | 17 | Transcriptional expression | *SWEET4* |
| *Os12g0159600* | Harpin-induced 1 domain containing protein | 16 | 0.997 | 13 | Transcriptional expression | *—* |
| *Os02g0769100* | Auxin responsive SAUR protein family protein | 15 | 0.996 | 6 | Transcriptional expression | *OsSAUR12* |

**Table S7** Transcription factor identification in five modules

|  | Families of development related transcription factors | | | | | | | | | | | Total number of TF related to development | Total number of TF in each module | Total number of genes in each module | Ratio |
| --- | --- | --- | --- | --- | --- | --- | --- | --- | --- | --- | --- | --- | --- | --- | --- |
|  | WRKY | AP2 | MYB | GRAS | C2H2 | MADS | GARP | NAC | SBP | bZIP | bHLH |  |  |  |  |
| black | 1 | 0 | 1 | 0 | 0 | 0 | 0 | 1 | 1 | 1 | 0 | 5 | 11 | 258 | 0.043 |
| brown | 4 | 9 | 8 | 1 | 6 | 4 | 5 | 1 | 0 | 7 | 5 | 50 | 92 | 2539 | 0.036 |
| yellow | 1 | 4 | 4 | 3 | 4 | 0 | 0 | 0 | 1 | 5 | 2 | 24 | 58 | 1389 | 0.042 |
| blue | 10 | 15 | 19 | 4 | 9 | 1 | 8 | 14 | 3 | 9 | 13 | 105 | 162 | 3245 | 0.050 |
| green | 7 | 12 | 12 | 3 | 6 | 3 | 9 | 6 | 2 | 7 | 10 | 77 | 129 | 1318 | 0.078 |
| Total number of TF in five modules | 23 | 40 | 44 | 11 | 25 | 8 | 22 | 22 | 7 | 29 | 30 |  |  |  |  |
| TF number of each family in rice | 128 | 180 | 185 | 67 | 139 | 88 | 71 | 165 | 28 | 110 | 181 |  |  |  |  |
| Ratio | 0.18 | 0.2 | 0.2 | 0.16 | 0.18 | 0.09 | 0.31 | 0.1 | 0.25 | 0.26 | 0.17 |  |  |  |  |

TF: Transcription Factor；The last column, Ratio = TF in each module/total number of module gene; The last row, Ratio= total number of TF families in 5 module/total number of TF families in rice (*Oryza sativa japonica*).

Table S8. Four GO terms likely to be involved in sterile lemma development

| **flower development** | | **plant organ formation** | | **reproductive  shoot system development** | | **regulation  of developmental process** | |
| --- | --- | --- | --- | --- | --- | --- | --- |
| *Os02g0686100* | RING-H2 zinc finger domain | *Os01g0510100* | belongs to the protein kinase superfamily(MKK6) | *Os02g0778200* | Belongs to the class-I aminoacyl-tRNA synthetase family | *Os01g0763200* | TCP family transcription factor |
| *Os02g0778200* | Belongs to the class-I aminoacyl-tRNA synthetase family | *Os03g0246900* | Lateral organ boundaries (LOB) domain | *Os02g0686100* | RING-H2 zinc finger domain | *Os01g0284500* | germin-like protein |
| *Os03g0856700* | Belongs to the iron ascorbate-dependent oxidoreductase family(*GA20ox1*) | *Os05g0455200* | Seed shattering;BEL1-type homeobox gene（*SH5*） | *Os03g0856700* | Belongs to the iron ascorbate-dependent oxidoreductase family(GA20ox1) | *Os01g0859500* | positive regulation of biological process |
| *Os04g0548700* | Homeobox associated leucine zipper | *Os08g0547300* | cytochrome p450 | *Os04g0580700* | MADS-box transcription factor 17 | *Os01g0831000* | Helix-loop-helix DNA-binding domain |
| *Os04g0569100* | GL2-type homeobox gene（*Rox4*） | *Os04g0580700* | K-box region | *Os04g0548700* | Homeobox associated leucine zipper | *Os01g0588200* | Mitochondrial outer membrane protein porin 3 |
| *Os04g0580700* | MADS-box transcription factor 17 | *Os01g0831000* | Helix-loop-helix DNA-binding domain | *Os04g0569100* | GL2-type homeobox gene（*Rox4*） | *Os02g0686100* | RING-H2 zinc finger domain |
| *Os05g0455200* | Seed shattering;BEL1-type homeobox gene（*SH5*） | *Os07g0669500* | Frizzy panicle; BRANCHED FLORETLESS 1; small grain and Dense panicle 7; ERF domain protein(*FZP*) | *Os05g0455200* | Seed shattering;BEL1-type homeobox gene（*SH5*） | *Os02g0161300* | SNF2 family N-terminal domain(BTAF1) |
| *Os06g0340600* | Phosphatidylinositol 3- and 4-kinase |  |  | *Os06g0340600* | Phosphatidylinositol 3- and 4-kinase | *Os03g0799600* | Bromo adjacent homology domain |
| *Os07g0108900* | MADS-box transcription factor 15 |  |  | *Os07g0160100* | YABBY protein | *Os04g0580700* | MADS-box transcription factor 17 |
| *Os07g0160100* | YABBY protein (*OsYABBY1*) |  |  | *Os07g0437000* | YUCCA-LIKE GENE 6; IAA synthetic pathway gene | *Os04g0569100* | GL2-type homeobox gene（*Rox4*） |
| *Os07g0437000* | *YUCCA-LIKE GENE 6*; IAA synthetic pathway gene |  |  | *Os07g0108900* | MADS-box transcription factor(AP1) | *Os05g0494600* | F-box-like |
| *Os07g0669500* | Frizzy panicle; BRANCHED FLORETLESS 1; small grain and Dense panicle 7; ERF domain protein(*FZP*) |  |  | *Os07g0669500* | Frizzy panicle; BRANCHED FLORETLESS 1; small grain and Dense panicle 7; ERF domain protein(*FZP*) | *Os06g0340600* | Phosphatidylinositol 3- and 4-kinase |
| *Os10g0564000* | KH domain |  |  | *Os10g0564000* | KH domain | *Os07g0577500* | Belongs to the ubiquitin-conjugating enzyme family |
|  |  |  |  |  |  | *Os07g0160100* | YABBY protein |
|  |  |  |  |  |  | *Os07g0437000* | YUCCA-LIKE GENE 6; IAA synthetic pathway gene |
|  |  |  |  |  |  | *Os07g0108900* | MADS-box transcription factor 15 |
|  |  |  |  |  |  | *Os07g0669500* | Frizzy panicle; BRANCHED FLORETLESS 1; small grain and Dense panicle 7; ERF domain protein(*FZP*) |
|  |  |  |  |  |  | *Os08g0547300* | cytochrome p450 |
|  |  |  |  |  |  | *Os09g0434500* | AP2 domain |
|  |  |  |  |  |  | *Os10g0564000* | KH domain |
|  |  |  |  |  |  | *Os12g0571100* | cellular response to zinc ion(MT4) |
|  |  |  |  |  |  | *Os12g0210500* | Auxin-Regulated Gene Involved in Organ(ARGOS) |
|  |  |  |  |  |  |  |  |

**Table S9** Primers for qRT-PCR

| **Primer Name** | **Sequence (5'→3')** | **MSU Locus/Gene Name** | **Use** |
| --- | --- | --- | --- |
| P450-F | ATTGGCTTCCCTTGTCAGATAA | *Os08g0547300/cytochrome P450* | qRT-PCR |
| P450-R | ACACATCTCAACAAGCACTAGA | *Os08g0547300/cytochrome P450* |
| OsIAA15-F | GACAAGTTCTTCTCCCACTTCA | *Os05g0178600/OsIAA15* |
| OsIAA15-R | AGAGCTTTTCATGAGACGAAGA | *Os05g0178600/OsIAA15* |
| OsGA20ox1-F | TACTGCCACGAGATGAGCC | *Os03g0856700/OsGA20ox1* |
| OsGA20ox1-R | CACGCCGGGTAGTAGTTGAG | *Os03g0856700/OsGA20ox1* |
| OsLBD37-F | CTAGGGTGGTTGGTTGTGATC | *Os03g0445700/OsLBD37* |
| OsLBD37-R | GGAGATGAAGGAGAGGAGTCC | *Os03g0445700/OsLBD37* |
| GRAS-F | GTTGCTGTAATGATCTCAGCTG | *Os10g0551200/GRAS* |
| GRAS-R | AGCTACTGTACCAGTACAACAC | *Os10g0551200/GRAS* |
| OSH1-F | AGGTAAACAACAAGGCACA | *Os03g0727000/OSH1* |
| OSH1-R | GCTCAAGACACGCAGGAT | *Os03g0727000/OSH1* |
| HL6-F | TTATGATAAGGAGGAGAAGGCC | *Os06g0657500/HL6* |
| HL6-R | CGTGCTGATGATGTCTTGTTAC | *Os06g0657500/HL6* |
| PLA3-F | ATAGAAAGACTTGCCAGAAC | *Os03g0790600/PLA3* |
| PLA3-R | AAATCTCCACAATAGCCAC | *Os03g0790600/PLA3* |
| Actin-F | AGTGTCTGGATTGGAGGAT | *Os03g0718100/ACT1* |
| Actin-R | TCTTGGCTTAGCATTCTTG | *Os03g0718100/ACT1* |

**Table S10** Primers for transient expression regulation assay

| **Primer Name** | **Sequence (5'→3')** | **MSU Locus/Gene Name** | **Use** |
| --- | --- | --- | --- |
| WGL9-G1-F | TGGGATCCCCGGGTGAGCTCATGTCGTCGTCGTCC | *Os07g0139300/G1* | Transient expression regulation assay |
| WGL9-G1-R | GCGGCCGCACTAGTAAGCTTTCAACTGAAGGTGTT | *Os07g0139300/G1* |
| LUC-GA20ox1pro-F | TATCGATAAGCTTGGAGGAAGGGAGGGTAGAAGAA | *Os03g0856700/* *OsGA20ox1* |
| LUC- GA20ox1pro-R | AATTCGATATCAAGCTTAATTGTTGATAATCTAGC | *Os03g0856700/* *OsGA20ox1* |
| LUC-IAA15pro-F | TATCGATAAGCTTGACTCAAAGGAAAGTTACCAAG | *Os05g0178600/OsIAA15* |
| LUC-IAA15pro-R | AATTCGATATCAAGCTCTCCGTCTCCACCGACATG | *Os05g0178600/OsIAA15* |
| WGL9-LBD37-F | TGGGATCCCCGGGTGAGCTCATGAGCTGCAACGGT | *Os03g0445700/OsLBD37* |
| WGL9-LBD37-R | GCGGCCGCACTAGTAAGCTTTCAGACAAAAAGGTT | *Os03g0445700/OsLBD37* |
| WGL9-OSH1-F | TGGGATCCCCGGGTGAGCTCATGGAGGAGATCTCC | *Os03g0727000/OSH1* |
| WGL9-OSH1-R | GCGGCCGCACTAGTAAGCTTCTAGCCGAGCCTGTA | *Os03g0727000/OSH1* |
