# Supplementary material for: Analysis of co-expression and gene regulatory networks associated with sterile lemma development in rice
Source: BMC Plant Biol. 2023 Jan 6;23:11. doi: 10.1186/s12870-022-04012-x (PMC9817312; doi:10.1186/s12870-022-04012-x)
Supplement: Supplementary file 5 — Additional file 5. [file 12870_2022_4012_MOESM5_ESM.pdf]

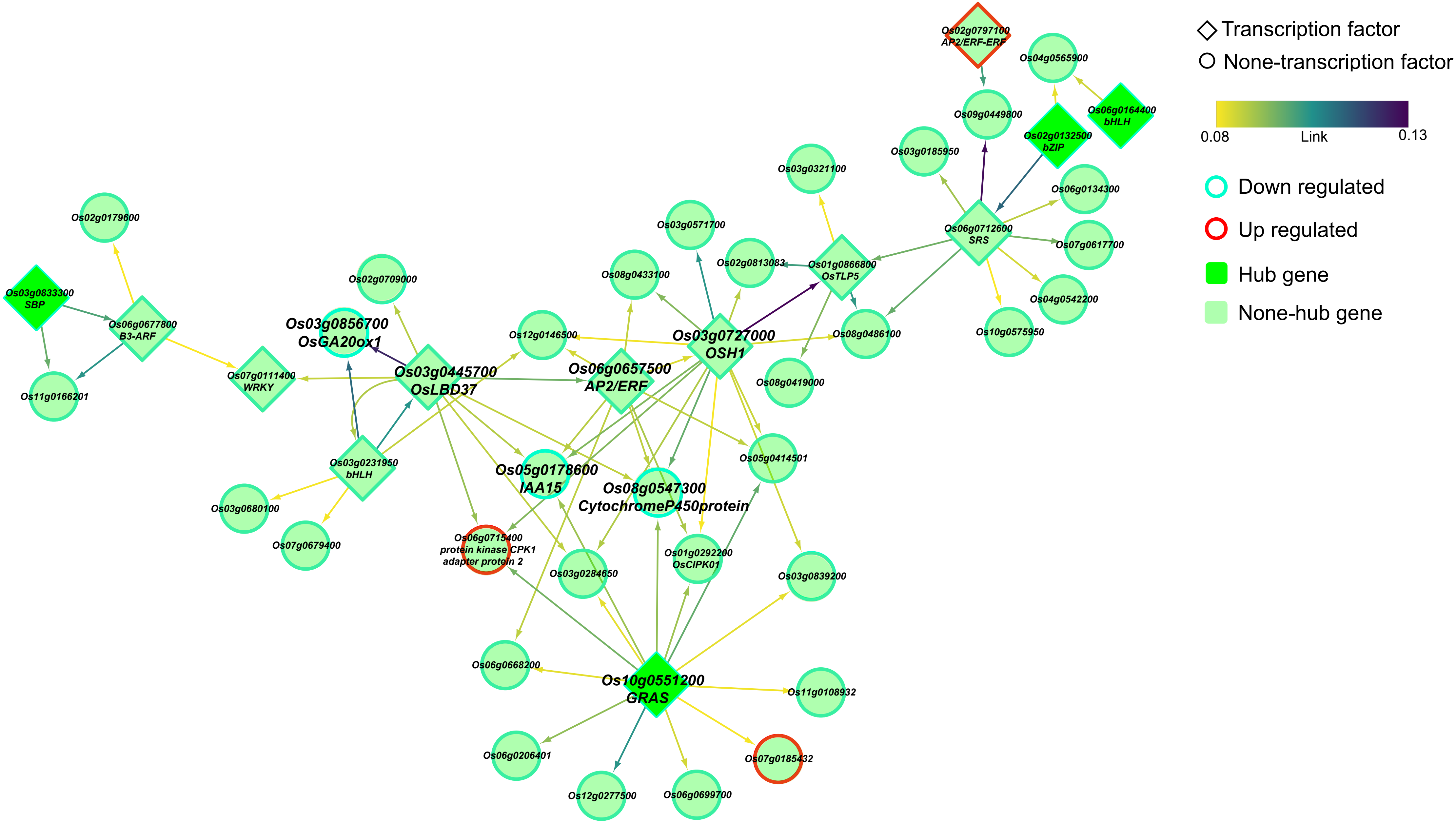

Fig. S13. The regulatory network in the green module.Transcription factors are annotated with symbols or family names; descriptions of target genes regulated by more than two transcription factors are annotated.
